# Identification of vaccine targets & design of vaccine against SARS-CoV-2 coronavirus using computational and deep learning-based approaches

**DOI:** 10.7717/peerj.13380

**Published:** 2022-05-19

**Authors:** Bilal Ahmed Abbasi, Devansh Saraf, Trapti Sharma, Robin Sinha, Shachee Singh, Shriya Sood, Pranjay Gupta, Akshat Gupta, Kartik Mishra, Priya Kumari, Kamal Rawal

**Affiliations:** Centre for Computational Biology and Bioinformatics, Amity Institute of Biotechnology, Amity University Uttar Pradesh, Noida, Uttar Pradesh, India

**Keywords:** Reverse vaccinology, Epitopes, Vaccine-designing, Deep learning, SARS-CoV-2, Molecular docking

## Abstract

An unusual pneumonia infection, named COVID-19, was reported on December 2019 in China. It was reported to be caused by a novel coronavirus which has infected approximately 220 million people worldwide with a death toll of 4.5 million as of September 2021. This study is focused on finding potential vaccine candidates and designing an *in-silico* subunit multi-epitope vaccine candidates using a unique computational pipeline, integrating reverse vaccinology, molecular docking and simulation methods. A protein named spike protein of SARS-CoV-2 with the GenBank ID QHD43416.1 was shortlisted as a potential vaccine candidate and was examined for presence of B-cell and T-cell epitopes. We also investigated antigenicity and interaction with distinct polymorphic alleles of the epitopes. High ranking epitopes such as DLCFTNVY (B cell epitope), KIADYNKL (MHC Class-I) and VKNKCVNFN (MHC class-II) were shortlisted for subsequent analysis. Digestion analysis verified the safety and stability of the shortlisted peptides. Docking study reported a strong binding of proposed peptides with HLA-A*02 and HLA-B7 alleles. We used standard methods to construct vaccine model and this construct was evaluated further for its antigenicity, physicochemical properties, 2D and 3D structure prediction and validation. Further, molecular docking followed by molecular dynamics simulation was performed to evaluate the binding affinity and stability of TLR-4 and vaccine complex. Finally, the vaccine construct was reverse transcribed and adapted for *E. coli* strain K 12 prior to the insertion within the pET-28-a (+) vector for determining translational and microbial expression followed by conservancy analysis. Also, six multi-epitope subunit vaccines were constructed using different strategies containing immunogenic epitopes, appropriate adjuvants and linker sequences. We propose that our vaccine constructs can be used for downstream investigations using *in-vitro* and *in-vivo* studies to design effective and safe vaccine against different strains of COVID-19.

## Introduction

**Figure 1:**
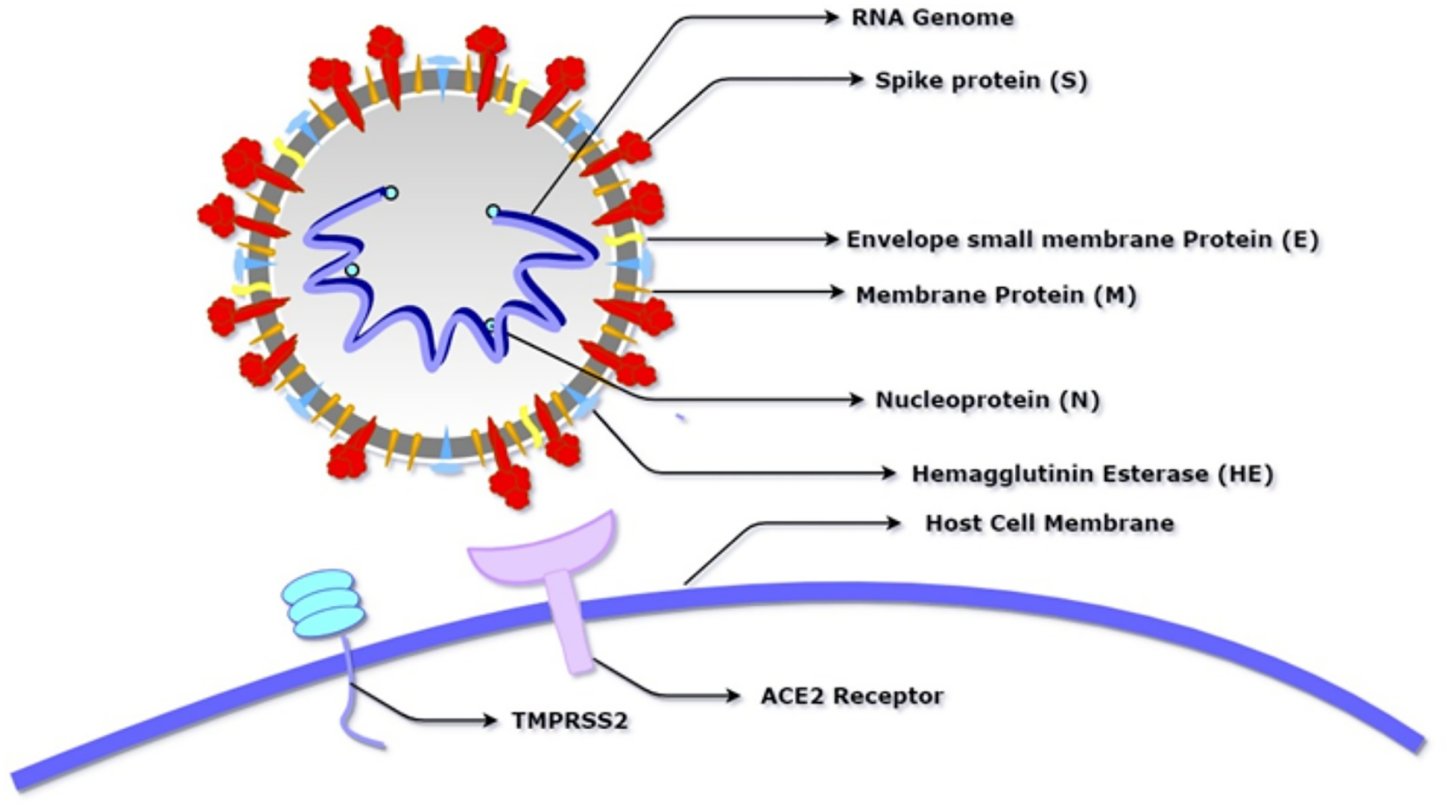
Schematic diagram of SARS-CoV-2 showing its basic component proteins along with its receptor binding site, angiotensin-converting enzyme 2 (ACE2) and transmembrane serine protease (TMPRSS2). The virus consists of a spherical membrane (shown in white and grey) which constitutes membrane protein (shown in orange), spike protein (shown in red), hemagglutinin esterase (shown in blue), and envelope small membrane protein (shown in yellow). The spike protein binds to the ACE2 receptor of the host cell after being activated by the proteolytic cleavage activity of TMPRSS2.

Coronavirus belongs to a large family of viruses called “Coronaviridae” (order Nidovirales) which are characterised by crown-like spikes on their surface and usually infect the respiratory system of humans and other vertebrates (Fig. 1). The epidemiological studies indicate the viral transmission from animal to human and thereafter from seeding clusters of human-human transmissions with the reproduction number (R_0_) ranges between 2.2–2.9 for humans (Lai et al., 2020). It can come under any of the four genera: Alphacoronavirus, Betacoronavirus, Gammacoronavirus, and Deltacoronavirus. The first incidence of human coronaviruses can be traced back to the mid-1960s. In the recent past, scientists have identified seven sub-types of the coronavirus that are known to cause infection in human beings. These include 229E (Alphacoronavirus); NL63 (Alphacoronavirus); OC43 (Betacoronavirus); HKU1 (Betacoronavirus); MERS-CoV (the Betacoronavirus that causes MERS); SARS-CoV (Betacoronavirus causing SARS) and SARS-CoV-2 (n-2019-CoV, Betacoronavirus). The first four viruses cause infection in the upper section of the respiratory tract that results in a mild infection while the other three viruses affect the lower section of the respiratory tract and result in severe respiratory syndrome in human beings (Centers for Disease Control and Prevention, 2020).

SARS-CoV-2 is the most recently evolved coronavirus that was first reported in Wuhan, China, which led to a mysterious pneumonia-like disease in humans and has been named COVID-19 by WHO. It has an incubation period of 4–7 days (Li et al., 2020). The pandemic, as of September 2021 has resulted in more than 220 million cases worldwide and a death toll of approximately 4.5 million. The worst hit nations are the USA, UK, Brazil, Italy, France, and Spain; all having crossed more than 20,000 deaths with the USA having more than 110,000 deaths (Worldometer, 2021, https://www.worldometers.info/coronavirus). The epidemiological studies have shown the Huanan seafood market to be the source of this outbreak, indicating an animal-to-human route, also known as zoonosis, as the prime transmission mode (Nishiura et al., 2020). Similar outbreaks in 2002–03 and in 2012 of Severe Acute Respiratory Syndrome (SARS) and Middle East Respiratory Syndrome (MERS), have shown a fatality rate of ~10% and ~35% respectively. SARS and MERS viruses were known to transmit from animal-to-human (Guo et al., 2020b). For this reason, extensive studies were conducted to understand the transmission of viral infections in humans and animals.

At the molecular level, coronaviruses are non-segmented, enveloped, positive, single stranded RNA viruses (~30 kb), having a 5′ cap and 3′ poly-A tail. This virus propagates by forming a replication-transcription complex (RTC) using its gRNA as a template. The RTC further encodes all the structural and non-structural proteins required for viral propagation. The viral genome is found to contain six ORFs. The first ORF (ORF1a/b) encodes 16 non-structural proteins and the rest encodes the four main structural proteins: spike (S), membrane (M), envelope (E) and nucleocapsid (N) (Spaan, Cavanagh & Horzinek, 1988; Chen, Liu & Guo, 2020). Presently, scientists have submitted 3,270,462 genomes of SARS-CoV-2 in Global Initiative on Sharing All Influenza Data (GISAID) and one of these has been released on GenBank with accession ID MN908947. In a recent phylogenetic study by Jiang, Du & Shi (2020), SARS-CoV-2 was found to be very similar to the bat SARS-like coronavirus, with 89% similarity at genomic level.

## Materials and Methods

### Data acquisition

Severe Acute Respiratory Syndrome Coronavirus-2 (SARS-CoV-2) isolate Wuhan-Hu-1, complete genome (accession ID NC_045512), and its coding sequences were retrieved from NCBI database in FASTA format (Agarwala et al., 2018). The crystal structures of human alleles, HLA-A*02 (PDB ID: 6O4Y) (Mishto et al., 2019) and HLA-B7 (PDB ID: 3VCL) (Brennan et al., 2012) were retrieved from Protein Data Bank (PDB) (Rose et al., 2015) to conduct the binding affinity studies with the predicted epitopes. HLA-A*02 was selected due to its presence in the majority of population in Wuhan region whereas HLA-B7 was selected as it is one of the predominant alleles in the world (Gonzalez-Galarza et al., 2019). Additionally, the peptide sequences of three different adjuvants were extracted from NCBI Database. These sequences includes L7/L12 50s ribosomal protein (accession ID WP_088359560.1, Flavobacteriaceae bacterium JJC), β-defensin and HABA proteins (accession ID AGV15514.1; *M. tuberculosis*).

### Workflow

**Figure 2:**
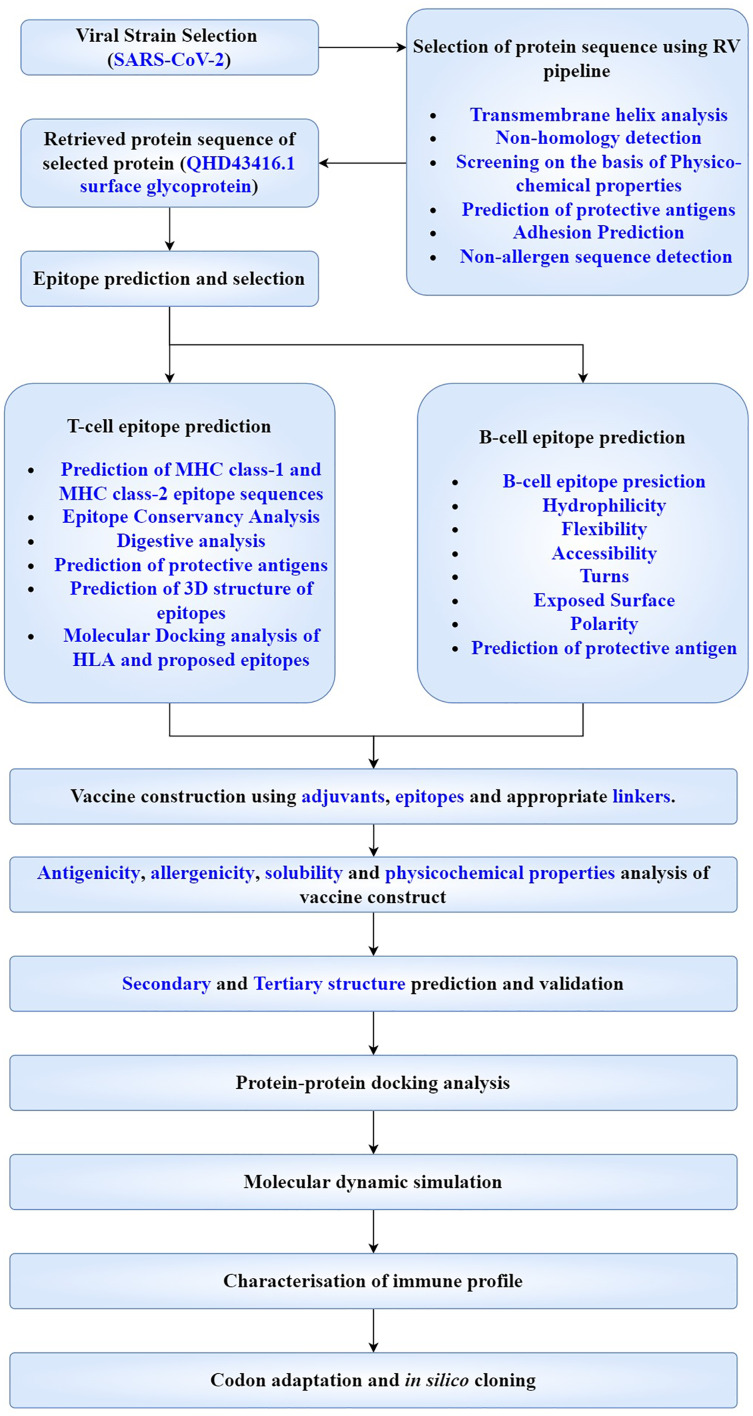
Flow chart depicting the multi-epitope subunit vaccine development against SARS-CoV-2.

Flow chart representation showing the workflow adopted has been made and the whole approach is summarised in subsequent sections (Fig. 2).

#### Identification of surface exposed proteins

Among the proteins encoded by pathogens, the surface and secretory proteins play important roles in the pathogenesis process, which include alterations in the host cell to the advantage of the pathogen, adhesion & invasion of host cell, host cell toxicity and defence against the host-immune response. Furthermore, the outer membrane proteins of the pathogen are involved in interactions with B-cells and Antigen Presenting Cells (APCs) (Hizbullah et al., 2018). These attributes of the surface and secretory proteins make them attractive drug & vaccine targets. Retrieval and selection of the outer cellular membrane proteins for the purpose of vaccine design and construction was performed using the state-of-the-art pipeline called VaxELAN (Rawal et al., 2021). The pipeline uses three different tools namely:- CELLO (Yu, Lin & Hwang, 2004), Virus-mPloc tool (Shen & Chou, 2010) and PSORTb (Yu et al., 2010) to determine the location of a given protein.

#### Trans-membrane (TM) analysis

Several studies have indicated that it is difficult to purify proteins with more than one transmembrane helix, so it seems reasonable to exclude these proteins from the selection process (He, Xiang & Mobley, 2010). Therefore, tools such as HMMTOP (Tusnady & Simon, 2001), TMHMM (https://services.healthtech.dtu.dk/service.php?TMHMM-2.0) and TMPred (Hofmann & Stoffel, 1993) were used to screen those proteins that have less than or equal to one transmembrane alpha-helices in their structure.

#### Non-homology analysis

Those viral proteins which are dissimilar to human proteins are considered to be good vaccine candidates since the vaccines based upon these proteins would minimize any kind of side-effect and cross-reactivity. To find such proteins, SARS-CoV-2 proteome was screened against the proteome of *Homo sapiens* (NCBI database) using the BLASTp tool (Altschul et al., 1990). The proteins having ≥35% identity, query coverage ≥35% and E value <10e-5 were filtered.

#### Physicochemical property analysis

With the help of ProtParam tool, various physicochemical properties of viral proteins were computed (Gasteiger, 2003). Based on these properties, those proteins were selected which were predicted to be stable in nature (*i.e*., instability index less than 40).

#### Antigenicity prediction

Doytchinova and Flower had proposed an alignment free approach in their VaxiJen v2.0 server, which is based on auto cross covariance transformation of protein sequences into uniform vectors of principal amino acid properties (Doytchinova & Flower, 2007). Using this approach, proteins whose antigenicity scores were greater than the threshold value of 0.4 were selected for further evaluation.

#### Adhesion prediction

Adhesin proteins play a significant role in the establishment of pathogen-based infections. Therefore, targeting the adhesin and adhesin-like proteins in vaccine development can help in combating such infections by blocking their function and preventing their adherence to the host cells (Wizemann, Adamou & Langermann, 1999). To achieve this objective, a tool named FungalRV with the threshold value of greater than or equal to −1.2 was employed (Chaudhuri et al., 2011). Though this tool was developed using proteins drawn from the fungal system, still, it provides a detailed analysis as well as clues for effective vaccine design.

#### Non-allergenicity analysis

Vaccines, just like drugs, also have the potential to cause allergic reactions. Therefore, it is important to check if the protein candidate acts as an allergen or not (Chung, 2014). In this study, a resource named AllergenOnline was used for the identification of proteins having potential allergenic action (Goodman et al., 2016). Here, only those proteins were selected which were labelled as non-allergen using BLASTp tool against the AllergenOnline database.

#### Evaluation of filtered protein

Physicochemical characterization of the shortlisted protein was performed using ProtParam and DiANNA (Ferre & Clote, 2005) tools. Protparam computes half-life, amino acid atomic composition, Grand average of hydropathicity (GRAVY), molecular weight and instability index. DiANNA is a neural network-based prediction system which was used to find the existence of disulphide-bonds in the viral proteins before subjecting them to B-cell and T-cell epitope predictions.

##### Linear B-cell epitope prediction

B-cell epitope prediction is performed to identify any surface-exposed regions in an antigen that can interact with an antibody. The primary sequence of the selected protein (QHD43416.1; spike S protein) was examined using BcePred server (Saha & Raghava, 2004) for prediction of continuous B-cell epitopes. Parameters including antigenicity, accessibility of surface, flexibility and hydrophilicity were also determined. Antigenic propensity and conservancy rate using IEDB Conservancy analysis tool (Bui et al., 2007) were also measured. Next, shortlisted epitopes were subjected for antigenicity evaluation using VaxiJen server.

##### T-cell epitope prediction

The T-cell epitope prediction was performed to identify those immunogenic peptides of an antigen that can stimulate CD4+ (HTL, Helper T-Lymphocyte) and CD8+ (CTL, Cytotoxic T-Lymphocyte) cells. T-cells operate by recognizing the antigen as peptides which are associated with major histocompatibility complex (MHC) molecules (Rötzschke et al., 1991). Cytotoxic T lymphocytes (CTL or CD8+ cells) curbs proliferation of antigens in the body by directly killing the viral infected cells or secreting antiviral cytokines. Tools such as ProPred-I (for MHC class-I alleles binding epitopes) (Singh & Raghava, 2003) and ProPred (for MHC class-II alleles binding epitopes) (Singh & Raghava, 2001) were used for T-cell epitope prediction. Using ProPred-I, filters were applied for proteasomal and immuno-proteasomal cleavages on the predicted MHC binding peptides (Sutmuller et al., 2001). Finally, only the high-scoring unique epitopes with 100% conservancy rates were considered in subsequent analysis. Furthermore, these epitopes were also subjected to toxicity analysis using the ToxinPred server (Gupta et al., 2013).

#### Structural modelling and molecular docking

Molecular docking is used to investigate the interaction of the predicted peptides with the MHC molecules using binding energies and contact residues (Rawal et al., 2019). With the help of PEP-FOLD (Maupetit, Derreumaux & Tuffery, 2009) server at RPBS MOBYLE (Neron et al., 2009) portal, the 3-D structure of the predicted peptides was determined. Next, 3D structures of human allele HLA A*02 (crystallized at the resolution of 1.58 Angstrom) was retrieved from PDB (ID: 6O4Y). Since allele HLA A*02, is found mostly in the population of Wuhan, therefore we used the 6O4Y structure for docking studies using the HPEPDOCK server (Zhou et al., 2018). HLA-B7 protein structures were also used for comparative studies.

#### Construction of final vaccine

**Figure 3:**
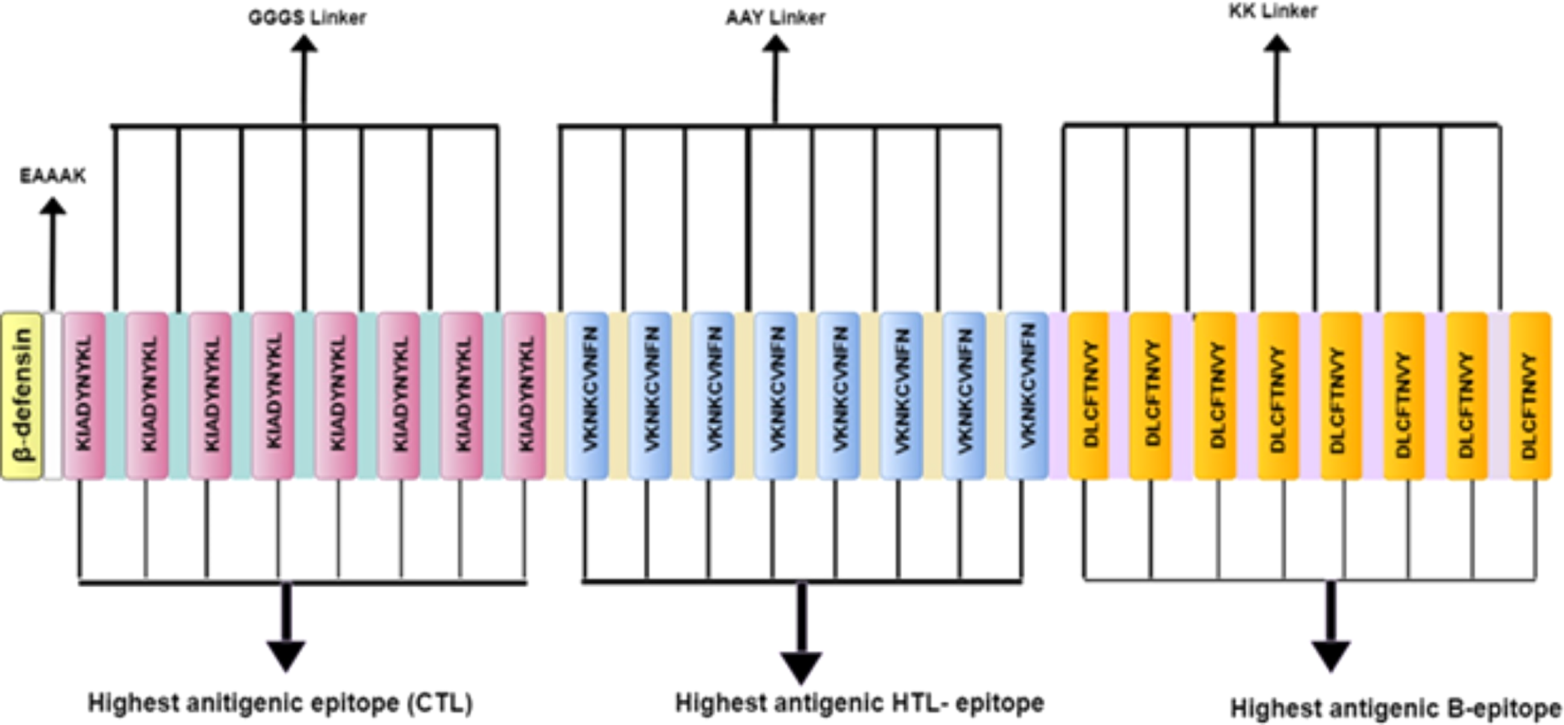
Schematic diagram of multi-epitope vaccine peptide. It is a 32 (insert amino acid number) amino acid long sequence having Beta-defensin as an adjuvant (light canary yellow) which is connected to the highest antigenic CTL epitope sequence (pink) through EAAAK linker (white). The CTL epitopes are linked to each other by GGGS linkers (grayish cyan), and to the highest antigenic HTL epitope (light blue) by AAY linkers (very soft yellow). Next, the HTL epitopes are linked to each other through AAY linkers, and to the highest antigenic B Epitope (vivid yellow) through KK linkers (pale violet). The B epitopes are linked to each other using the KK linkers as well.

Six potential multi-subunit vaccines against COVID-19 were constructed by using high-scoring CTLs, HTLs, and B-cell epitopes. The immunogenic peptides of length 9–12 amino acids were obtained from the shortlisted spike protein and merged together to formulate the vaccine candidates using distinct strategies. To differentiate between various constructs, the constructed vaccine sequences were labelled as V1, V2, V3, V4, V5 and V6. The strategy for constructing V1, V2 and V3, has been discussed in this section while the strategies of V4–V6 constructs is described in the (Supplemental File A). Each sequence starts from a distinct adjuvant sequence namely β-defensin, L7/L12 50s ribosomal protein and HABA protein, respectively. Each of these adjuvants have been reported to accentuate protective immune response (Meza et al., 2017). The adjuvant was linked to the first CTL epitope *via* EAAAK linker, and all the CTL epitope repeats were linked with each other by the GGGS linker. Conjugation of the CTL epitope with the HTL epitope and the HTL epitope repeats among themselves was carried out using AAY linker, whereas conjugation of the HTL epitope with the B-cell epitope and B-cell epitope repeats among themselves was performed using the KK linker (Fig. 3). To determine the order of different components in the vaccine construct, information previously reported in studies namely Ebola virus (Ullah, Sarkar & Islam, 2020), Avian influenza A (H7N9) (Hasan et al., 2019b), Monkeypox virus (Farjana, Islam & Taiebah, 2020) and *Marburg marburgvirus* (Hasan et al., 2019a) was utilised.

#### Antigenicity, allergenicity, solubility and physicochemical analysis of vaccine constructs

Antigenicity of vaccine constructs or chimeric protein was evaluated using the Vaxijen v2.0 server with a threshold of 0.4. Further, non-allergenic nature of all the constructs was evaluated using Algpred (Saha & Raghava, 2006a). This tool incorporates methods based on SVM, motif searching, and BLAST searches on allergen representative peptides (ARPs). The solubility of these constructs was also determined using Protein-Sol (Hebditch et al., 2017). Moreover, various physicochemical properties of the vaccine constructs were also determined using the ProtParam tool including their isoelectric pH, GRAVY values, molecular weight, instability index, estimated half-life, and aliphatic index.

#### Secondary and tertiary structure prediction

Secondary structures of the constructed vaccines were obtained using PSIPRED (McGuffin, Bryson & Jones, 2000) server and the structural composition was determined by employing CFSSP server (Ashok Kumar, 2013). The tertiary structure of the vaccine constructs was predicted by using the I-TASSER server (Zhang, 2008).

#### 3D structure refinement and validation

The I-TASSER server predicted the 3-D model of the vaccine construct. It is based on a hierarchical approach for predicting high resolution protein structure and function. Among all the predicted 3D models of a vaccine construct, the model having the highest C-score was selected. Refinement of the predicted model was performed to improve its accuracy by using online refinement tools namely, ModRefiner (Xu & Zhang, 2011) and 3Drefine (Bhattacharya et al., 2016). The refined protein structure was further validated using the Ramachandran plots generated by online tool RAMPAGE (https://bio.tools/RAMPAGE).

#### Prediction of discontinuous B-cell epitopes

In order to remove infections from the body, antibodies must recognise and interact with antigenic epitopes. Almost 90% of B-cell epitopes are discontinuous or conformational in nature. This means that the amino acid residues comprising the epitope are remotely located in the primary chain and are brought in close proximity as a result of protein folding. Ellipro available at http://tools.iedb.org/ellipro/ was used for identification of conformational epitopes (Ponomarenko et al., 2008). This tool assumes that residues protrude from protein surface are more accessible for antibody binding.

#### Molecular docking of subunit vaccine with immune receptor

Molecular docking analysis is an essential tool for determining the interaction between a receptor and ligand molecule. The binding affinity of the vaccine construct with human toll-like receptors (TLR-8) was determined *via* several online docking servers. These servers include the HDOCK server (Yan et al., 2017), ClusPro 2.0 server (Kozakov et al., 2017) and PatchDock (Schneidman-Duhovny et al., 2005). The models obtained by PatchDock were further refined by FireDock (Andrusier, Nussinov & Wolfson, 2007). The crystal structure of TLR-8, obtained from RCSB protein data bank (PDB id: 3W3M, resolution: 2.70 Å) (Tanji et al., 2016), were used to analyse a desirable protein-protein complex in terms of better electrostatic interaction and binding energy.

#### Molecular dynamics simulation

The MD simulation was performed using GROMACS 2020.2 software based on Newtonian laws of atomic and molecular motion. It predicts the behaviour of ligands and receptor over a specific period of time. The OPLS-AA force-field was used to prepare the input structure (Jorgensen, Maxwell & Tirado-Rives, 1996). The surface charge of the structure was further neutralized using Sodium and Chloride ions. GMX solvate was used to add the water molecules using TIP3P water molecule with a thickness of 10 Å (Harrach & Drossel, 2014). Further, to eliminate steric clashes, van der Waals interaction and hydrogen bonds forming between the water and complex molecules, the energy of the structures was minimized using steepest descent methods. Afterward, the system temperature was steadily increased from 0 to 300 K in a constant volume for 200 ps, and the system was then equilibrated at a constant pressure. Finally, the root-mean-square deviation (RMSD) and root-mean-square fluctuation (RMSF) of the ligand and the receptor were calculated over a 50-ns timeframe.

#### Characterisation of immune profile of the construct

To simulate the real response of an immune system to our final vaccine construct, the C-immSim immune server was employed (Castiglione & Bernaschi, 2004). This is a freely accessible web-server (http://150.146.2.1/C-IMMSIM/index.php) that works on the basis of Position-Specific Scoring Framework (PSSM) to simulate and predict immune interactions along with immunogenic epitopes. The tool was run on default parameters with time step of injection being 1, 42, 84 *i.e*., three times and vaccine injection without lipopolysaccharide.

#### Codon adaptation and *in silico* cloning of the chimeric protein

The Java Codon Adaptation Tool or JCat server was employed (http://www.jcat.de/) for the purpose of *in silico* codon adaptation in model organism *E. coli* strain K12 for the expression of protein vaccine (Grote et al., 2005). Vaccine constructs were reverse transcribed to possible DNA sequence and filters were applied to avoid rho-independent transcription termination, prokaryotic ribosome binding sites and cleavage sites of various restriction enzymes (BamHI and XhoI). The reverse-transcribed DNA sequence (RT-DNA) thus obtained is conjugated with XhoI and BamHI restriction sites at N-terminal and C-terminal sites, respectively. Next this adapted DNA sequence is incorporated into the multiple cloning site (MCS) of pET-28a (+) vector using the SnapGene tool (GSL Biotech, 2020).

### Evaluation of vaccine construct against other SARS-CoV-2 variants

SARS-CoV-2 is rapidly evolving virus as it has a very high mutation frequency. Throughout the pandemic, different genetic variants emerged around the world, to the horrors of humans, each posing a greater challenge for the nations to control the spread of disease. This is also a major issue from the vaccinology point of view as viability of existing vaccines needs to be continuously evaluated against different variants. Furthermore, it became necessary for the novel vaccine candidates developed by various researchers to cater to the problem of viral mutants. For this we evaluated the conservancy of our selected epitopes comprising the vaccine candidate against the surface glycoprotein (spike, S) sequences from various variants (Saba et al., 2021). For this, surface glycoprotein sequences of variants of concern {having Pango lineages: B.1.1.7, B.1.351, B.1.351.2, B.1.351.3, Delta variant (B.1.617.2, AY.1, AY.2, AY.3) P.1, P.1.1 and P.1.2} and variants of interest {having Pango lineages: B.1.427, B.1.429, B.1.525, B.1.526, B.1.617.1, B.1.617.3 and P.2} (https://www.cdc.gov/coronavirus/2019-ncov/variants/variant-info.html) were retrieved from NCBI virus portal (https://www.ncbi.nlm.nih.gov/labs/virus/vssi/#/) such that no protein sequence has any ambiguous character *i.e*., X for proteins (Saba et al., 2021). After this, S-Surface Glycoprotein (accession no.: QHD43416.1) was screened against the retrieved sequences of each variant using BLAST+ (Camacho et al., 2009) and best matching sequence for each variant was identified. After this, multiple sequence alignment (options; align by MUSCLE and algorithm: Neighbour Joining) and phylogenetic tress construction (using the Neighbour Joining method and substitution model used: p-distance) of identified best matches of all variants and QHD43416.1 were performed using MegaX (Kumar et al., 2018). This was followed by conservancy analysis of our identified BCL, HTL and CTL epitope (DLCFTNVY, VKNKCVNFN and KIADYNYKL respectively) using IEDB conservancy analysis tool (Bui et al., 2007).

### AI in potential vaccine detection

A total of 100 proteins were extracted and labelled as positive dataset-which were reported to be antigenic candidates using text mining and deep curation strategies (Jagannadham et al., 2016). Similarly, various control datasets, labelled as negative datasets were constructed which consist of proteins not known to produce any immune response in the host system. Subsequently, several bioinformatics, reverse vaccinology and immunoinformatics tools such as PSORTb, FungalRV, SignalP, TargetP, IEDB, BLASTp, ProtParam, Vaxijen, etc. were utilised to characterise proteins into positive and negative datasets. Thereafter, distributions of scores as well as ROC curves were generated to determine the cut-off. Further, each protein was converted into a feature vector. Next, the data was normalised using min-max normalisation function. This step was followed with training of the algorithm on two datasets: Model-1 was trained on viral proteins and Model-2 was trained on bacterial proteins. Thereafter, an LSTM network was constructed which consisted of two LSTM nodes, along with two fully connected nodes with leaky Relu activation function and a single fully connected node with sigmoid function as an output layer. Each hidden node in the network has weight and bias maximum normalize constraint of value 3 and was regularized using L2 regularization function to prevent overfitting during training. Cross validation was performed, and the dataset was divided into two parts. The first part had 170 equally weighted examples as used during training and 30 examples were used in testing or cross validation purposes. Our group has recently deployed a user-friendly cloud-based Vaxi-DL server for potential vaccine prediction (Rawal et al., 2022).

### Viral-host protein interactions

The interactions of spike glycoprotein with other host proteins were also investigated using String (v11.0 protein-protein interaction database) (Szklarczyk et al., 2015). Since SARS-CoV-2 interaction data was not available, the data derived from Coronavirus 229E (NCBI taxonomy ID: 11137), Human SARS coronavirus (NCBI taxonomy ID: 694009) and *Homo sapiens* (host) (NCBI taxonomy ID: 9606) was used.

## Results

In this study, several computational strategies such as reverse vaccinology, deep learning and immunoinformatics tools were used to find the most suitable protein vaccine candidate against SARS-CoV-2. Using the above-mentioned approaches, a protein named as Surface glycoprotein was shortlisted, and B-cell and T-cell epitopes were predicted for the construction of an epitope-based vaccine.

### Reverse vaccinology pipeline

Out of the 10 proteins of SARS-CoV-2, the integrated pipeline shortlisted one protein (Spike S-Surface Glycoprotein with accession ID QHD43416.1) as a potential vaccine candidate. The physicochemical properties of this protein were predicted by ProtParam and DiANNA (Table 1). The description of secondary structure was predicted by PSIPRED (Table 2).

**Table 1:** Physicochemical properties of QHD43416.1 surface glycoprotein (Severe Acute Respiratory Syndrome Coronavirus 2) as predicted by ProtParam and DiANNA.

Property	Value
Number of amino acids	1,273
Molecular weight	141,178.47
Theoretical pI	6.24
Atomic composition	Carbon (C) 6336, Hydrogen (H) 9770, Nitrogen (N) 1656, Oxygen (O) 1894, Sulphur (S) 54
Total number of negatively charged residues (Asp + Glu)	110
Total number of positively charged residues (Arg + Lys)	103
Formula	C_6336_H_9770_N_1656_O_1894_S_54_
Extinction coefficients	148,960 (assuming all pairs of Cys residues form cystines)
Estimated half-life	30 h
Instability index	33.01 (stable)
Aliphatic index	84.67
Grand average of hydropathicity (GRAVY) value	−0.079
Cysteine disulphide bond score	40

**Table 2:** Description of secondary structure as predicted by PsiPred.

Structural element	Percent composition
Strand	10
Helix	20
Coil	30
Disordered	40

### Recognition of B-cell epitopes

B-cell epitopes play a crucial role in the activation of B-cell mediated immune response against viral infections. The BcePred server was utilised to predict the continuous B cell epitopes. A total of 41 B-cell epitope sequences were predicted using BcePred. Physicochemical parameters like hydrophilicity (Parker, Guo & Hodges, 1986), exposed surface (Janin et al., 1978), turns (Pellequer, Westhof & Van Regenmortel, 1993), accessibility (Emini et al., 1985), flexibility (Karplus & Schulz, 1985) and antigenic propensity (Kolaskar & Tongaonkar, 1990) were also evaluated for prediction of linear epitopes. Furthermore, the IEDB conservancy tool was used to evaluate the predicted epitopes. Out of these, only 15 peptides were predicted to be highly antigenic in nature determined by the Vaxijen server. For instance, a peptide “DLCFTNVY” is predicted to be the highest-ranking peptide (with a score: 1.85) amongst the other shortlisted peptides (Table 3). Additionally, we have compared the shortlisted epitope with various prediction servers namely, BepiPred 2.0 (Jespersen et al., 2017), ABCpred (Saha & Raghava, 2006b) and the DLBEpitope server (Liu, Shi & Li, 2020) (Supplemental File SF, SF1).

**Table 3:** B-cell epitopes present on surfaces predicted *via* BCPRED.

S. No.	Antigenic propensity	Antigenic score
1.	DLCFTNVY	1.85
2.	YYVGYLQPR	1.46
3.	EPVLKGVKLHYT	1.41
4.	LIDLQEL	1.39
5.	TEILPVS	1.26
6.	EILDITPCSFGGVSVITPG	1.13
7.	SVVNIQK	1.08
8.	YQPYRVVVLSFELLH	0.97
9.	PHGVVFLHVTYVP	0.93
10.	YNYLYRLFR	0.86
11.	ECSNLLLQYGSFC	0.86
12.	MFVFLVLLPLVSSQCVNLTT	0.83
13.	LEPLVDLPIGI	0.82
14.	FNCYFPLQSY	0.82
15.	FSTFKCYGVSPT	0.8

### Recognition of T-cell epitopes

#### MHC-I allele binding T-cell epitopes

The ProPred-I tool was used to predict MHC-I binding T-cell epitopes (Table 4). Using Proteasome and ImmunoProteasome filters set at the threshold of 5%, all alleles were selected and only the top 10 peptides were chosen to be displayed by the ProPred-I server result. Only peptides with 100% conservancy rate were considered. Out of the 46 predicted MHC class-I binding epitopes, 45 epitopes were found to be conserved. For instance, we found that KIADYNYKL has the highest antigenicity score of 1.66 and binds to a number of alleles including HLA-A2, HLA-A*0201, HLA-A*0205, HLA-A3, HLA-B*0702 (Supplemental Table S1). Physicochemical properties of top eight selected epitopes were obtained by ToxinPred (Supplemental Table S2). Additionally, we have compared the shortlisted epitope with various prediction servers namely; CTLPred (Bhasin & Raghava, 2004), EpiJen (Doytchinova, Guan & Flower, 2006), NetCTL 1.2 server (Larsen et al., 2007), NetMHCpan-4.1 (Reynisson et al., 2020a) and Tepitool (Paul et al., 2016) (Supplemental File SF, SF2).

**Table 4:** List of top scoring MHC class I and MHC class II binding T-cell epitopes.

S. No.	MHC class I binding (CTL) epitopes	MHC class II binding (HTL) epitopes
1.	KIADYNYKL	VKNKCVNFN
2.	VVVLSFELL	YRFNGIGVT
3.	TLDSKTQSL	VVFLHVTYV
4.	GKQGNFKNL	FKCYGVSPT
5.	VRDLPQGFS	VNLTTRTQL
6.	PWYIWLGFI	IGINITRFQ
7.	NFGAISSVL	LVKNKCVNF
8.	QGFSALEPL	VVIGIVNNT

#### MHC-II allele binding T-cell epitopes

The ProPred tool was used to predict the MHC-II binding T-cell epitopes (Table 4). Among the 94 predicted epitopes, 90 were found to have 100% conservancy rate. Out of which, VKNKCVNFN was found to have the highest antigenicity score of 2.05 and binds to several alleles (Supplemental Table S3). The physicochemical properties of top eight selected epitopes were obtained by ToxinPred (Supplemental Table S4). Additionally, we have compared the shortlisted epitope with various prediction servers namely; IEDB MHC-II server (Wang et al., 2010), NetMHCIIpan 4.0 server (Reynisson et al., 2020b) and EpiTOP (Dimitrov et al., 2010) (Supplemental File SF, SF3).

### Structural modelling and molecular docking

**Figure 4:**
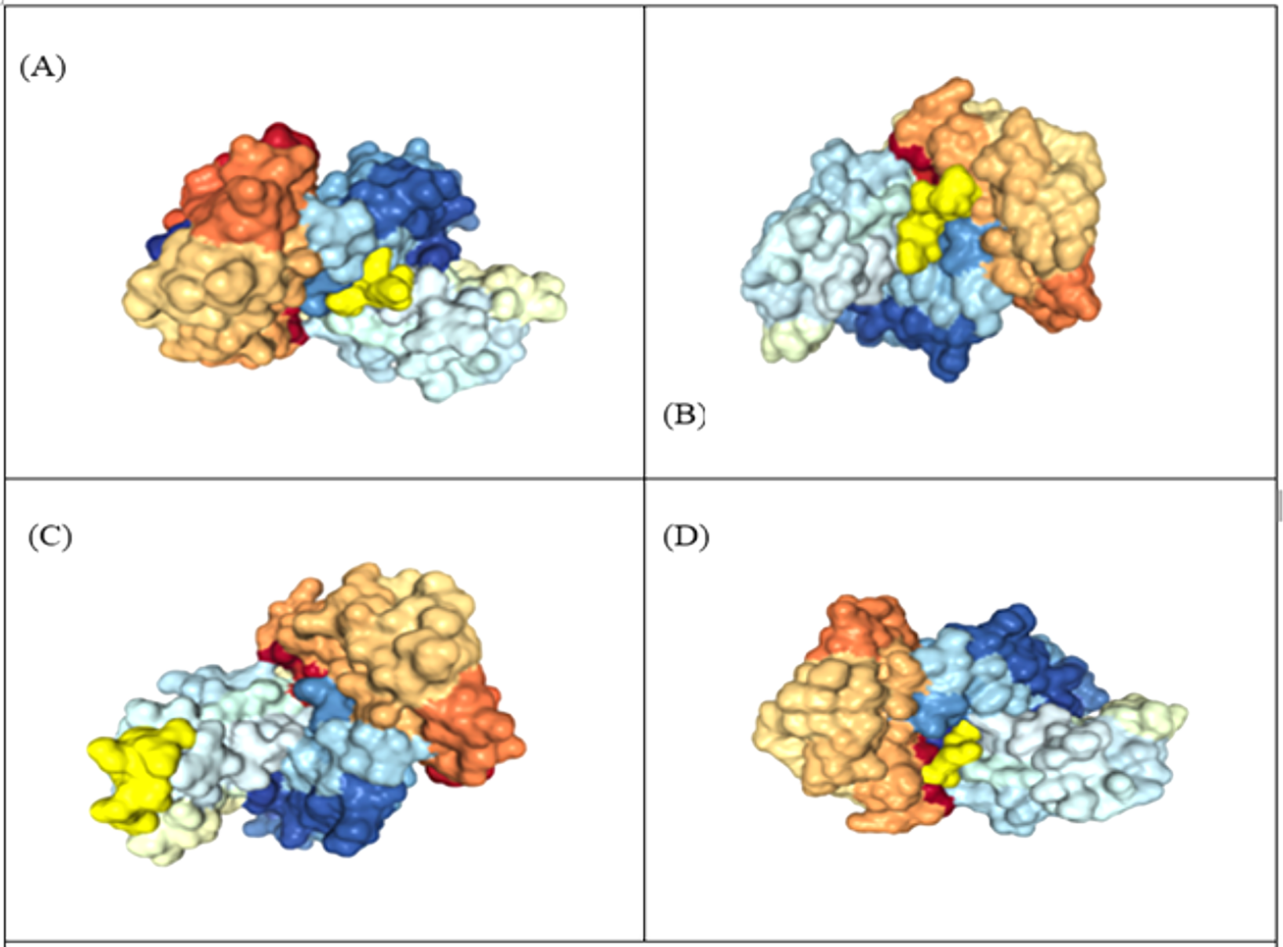
Representation of protein-peptide docked complex of top four MHC class-1 epitopes sequences. (A) KIADYNYKL, (B) VVVLSFELL, (C) TLDSKTQSL and (D) GKQGNFKNL, shown in golden yellow) in association with the HLA-A*02 allele using HPEPDOC. The epitopes have a binding affinityof −205.89, −157.77, −150.05 and −178.48 respectively with HLA_A*02.

The 3D structure of MHC class-I epitopes was predicted using PEP-FOLD. Molecular docking is a vital tool to understand protein-peptide interaction. Top four antigenic CTL epitopes: KIADYNYKL, VVVLSFELL, TLDSKTQSL and GKQGNFKNL were docked against various Human Leukocyte Antigen (HLA) using the web HPEPDOCK server under default settings to find their binding affinities. The epitopes have a binding affinity of −205.89, −157.77, −150.05 and −178.48 kcal mol^−1^ respectively with HLA-A*02 and −171.84, −166.31, −136.22 and −152.84 kcal mol^−1^ respectively with HLA-B7 (Table 5, Fig. 4).

**Table 5:** Protein-Peptide docking using web server HPEPDOCK of MHC-I with crystal structure of HLA*A2 and HLA*B7.

**Peptide**	**Human allele (PDB ID)**	**Docking score**
		**(kcal mol-1)**
For MHC-Class I with HLA-A*02		
KIADYNYKL	6O4Y	−205.89
VVVLSFELL	6O4Y	−157.77
TLDSKTQSL	6O4Y	−150.05
GKQGNFKNL	6O4Y	−178.48
**For MHC-Class I with HLA-B7**		
KIADYNYKL	3VCL	−171.84
VVVLSFELL	3VCL	−166.31
TLDSKTQSL	3VCL	−136.22
GKQGNFKNL	3VCL	−152.84

### Construction of vaccine

High scoring Linear B cell, CTL and HTL epitopes were used to construct multi epitope vaccines. Adjuvant sequences were used for enhancing immune interaction by utilizing its advantageous feature to act as an agonist and perform a significant part in improving the efficacy of vaccines (Marciani, 2003) (Supplemental File A).

### Antigenicity, allergenicity, solubility and physicochemical analysis of vaccine constructs

Vaccine construct V1 was predicted to be highly antigenic (Score 1.16 using the Vaxijen web server) (Table 6). In addition, V1 was also predicted to be non-allergenic by the Algpred tool. The solubility value of V1 was estimated to be 0.71 by Protein-sol tool (threshold value of 0.45) indicating that the constructed vaccine is more soluble than average soluble *E. coli* protein from the hypothetical dataset utilised by that tool. The molecular weight of the construct V1 with beta-defensin as an adjuvant (326 amino acids) was estimated to be 36.83 kDa with a theoretical isoelectric point value (pI) of 9.58. The half-life was estimated at 30 h in mammalian reticulocytes *in vitro*, and more than 20 h in yeast and more than 10 h in *E. coli in vivo*. The instability index (II) was estimated at 9.76, indicating that the vaccine is stable (Threshold II less than 40 indicates stability). The predicted aliphatic index was calculated to be equal to 67.61, indicating the thermostability of the proposed vaccine (Ikai, 1980). The predicted hydropathicity came to be −0.467 which denotes the vaccine construct V1 is hydrophilic in nature and can bind with molecules of water (Ali et al., 2017). The information regarding these parameters for remaining constructs can be retrieved from (Supplemental File B).

**Table 6:** Protein sequence of vaccine constructs V1, V2 and V3 along with their antigenicity analysis.

Vaccine construct	Composition/order	Sequence	Antigenicity score (Threshold = 0.4)
V1	Predicted CTL, HTL & BCL epitopes of spike Glycoprotein with β defensin adjuvant	GIINTLQKYYCRVRGGRCAVLSCLPKEEQIGKCSTRGRKCCRRKKEAAAKKIADYNYKLGGGSKIADYNYKLGGGSKIADYNYKLGGGSKIADYNYKLGGGSKIADYNYKLGGGSKIADYNYKLGGGSKIADYNYKLGGGSKIADYNYKLAAYVKNKCVNFNAAYVKNKCVNFNAAYVKNKCVNFNAAYVKNKCVNFNAAYVKNKCVNFNAAYVKNKCVNFNAAYVKNKCVNFNAAYVKNKCVNFNKKDLCFTNVYKKDLCFTNVYKKDLCFTNVYKKDLCFTNVYKKDLCFTNVYKKDLCFTNVYKKDLCFTNVYKKDLCFTNVY	1.16
V2	Predicted CTL, HTL & BCL epitopes of spike Glycoprotein with L7/L12 ribosomal protein adjuvant	MSDINKLAETLVNLKIVEVNDLAKILKEKYGLDPSANLAIPSLPKAEILDKSKEKTSFDLILKGAGSAKLTVVKRIKDLIGLGLKESKDLVDNVPKHLKKGLSKEEAESLKKQLEEVGAEVELKEAAAKKIADYNYKLGGGSKIADYNYKLGGGSKIADYNYKLGGGSKIADYNYKLGGGSKIADYNYKLGGGSKIADYNYKLGGGSKIADYNYKLGGGSKIADYNYKLAAYVKNKCVNFNAAYVKNKCVNFNAAYVKNKCVNFNAAYVKNKCVNFNAAYVKNKCVNFNAAYVKNKCVNFNAAYVKNKCVNFNAAYVKNKCVNFNKKDLCFTNVYKKDLCFTNVYKKDLCFTNVYKKDLCFTNVYKKDLCFTNVYKKDLCFTNVYKKDLCFTNVYKKDLCFTNVY	1.03
V3	Predicted CTL, HTL & BCL epitopes of spike protein with HABA adjuvant	MAENPNIDDLPAPLLAALGAADLALATVNDLIANLRERAEETRAETRTRVEERRARLTKFQEDLPEQFIELRDKFTTEELRKAAEGYLEAATNRYNELVERGEAALQRLRSQTAFEDASARAEGYVDQAVELTQEALGTVASQTRAVGERAAKLVGIELEAAAKKIADYNYKLGGGSKIADYNYKLGGGSKIADYNYKLGGGSKIADYNYKLGGGSKIADYNYKLGGGSKIADYNYKLGGGSKIADYNYKLGGGSKIADYNYKLAAYVKNKCVNFNAAYVKNKCVNFNAAYVKNKCVNFNAAYVKNKCVNFNAAYVKNKCVNFNAAYVKNKCVNFNAAYVKNKCVNFNAAYVKNKCVNFNKKDLCFTNVYKKDLCFTNVYKKDLCFTNVYKKDLCFTNVYKKDLCFTNVYKKDLCFTNVYKKDLCFTNVYKKDLCFTNVY	0.98

### Secondary structure prediction

**Figure 5:**
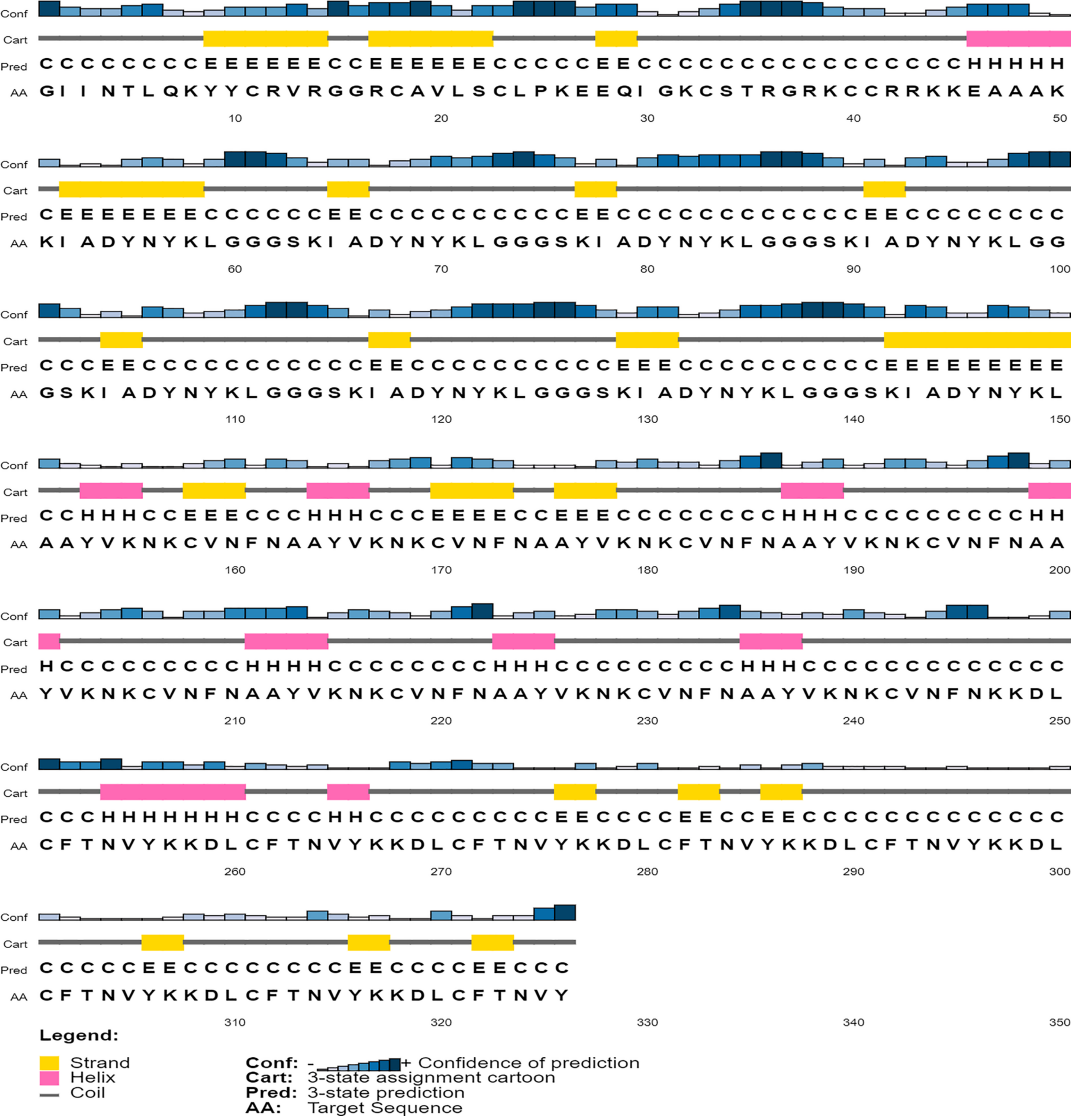
Graphical representation of secondary structure features of proposed subunit vaccine sequence using the PSIPRED tool.

The secondary structure of the vaccine construct V1 was predicted by PSIPRED (Fig. 5). It was predicted to have 62.9% helix, 29.8% beta-sheets and 12.6% turns by using the CFSSP tool (For secondary structure of V2–V6, see Supplemental File C).

### Tertiary structure prediction, refinement and validation

**Figure 6:**
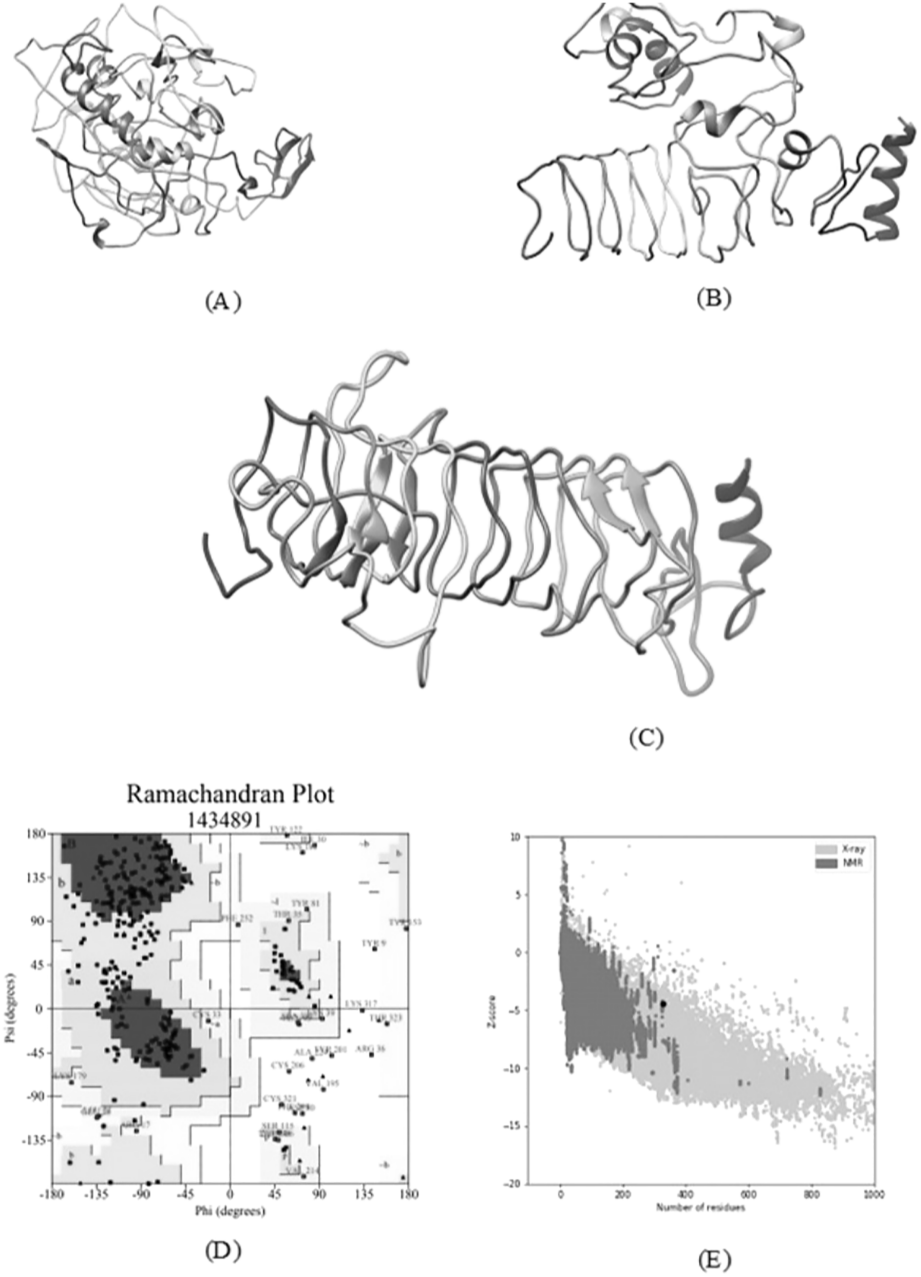
Tertiary structure modeling, refinement and validation. (A) The final 3D model of multi epitope vaccine chimeric protein generated *via* homology modelling on I-TASSER, (B) Refined model obtained *via* ModRefiner, (C) The refined 3D structure generated by 3DRefine (D) Ramachandran Plot Analysis signifying 57.0%, 38.9% and 4.0% of protein residues in favoured, allowed and disallowed (outlier) regions respectively, (E) ProSA-web, giving a Z-Score of −4.4.

A total of five 3D models (tertiary structures) of the vaccine construct V1 were predicted by I-TASSER server based on 10 best threading templates namely, 6buaA, 3du1X, 1kj6, 1kj6, 4plaA, 1kj6, 2xtwA, 1kj6A, 1kj6A and 4n9nA as identified by LOMETS (Wu & Zhang, 2007) from the PDB library. These best templates were selected from the LOMETS threading programs using the Z-score values during the I-TASSER modelling. The five models thus predicted had C-score values ranging between −3.36 and −4.19. Since the C score normally ranges from −5 to 2, with a higher value indicating higher confidence, the model with the highest C-score (Model 4 in case of vaccine construct V1 has highest C-score of −3.36) was chosen for further refinement by online refinement tool ModRefiner followed by 3Drefine. The refined 3D models of all vaccine constructs were validated by referring to the Ramachandran plot generated using RAMPAGE **(**http://mordred.bioc.cam.ac.uk/~rapper/rampage.php). The Ramachandran plot assessment of V1 predicted 73.8%, 18.8% and 7.4% residues to be in favoured, allowed and outlier regions, respectively (Fig. 6).

### Prediction of discontinuous B-cell epitopes

Ellipro predicted the four discontinuous B-cell epitopes and confirmed the presence of 210 residues among them with score ranging from 0.60 to 0.98 (Supplemental File D).

### Molecular docking of subunit vaccine with immune receptor

**Figure 7:**
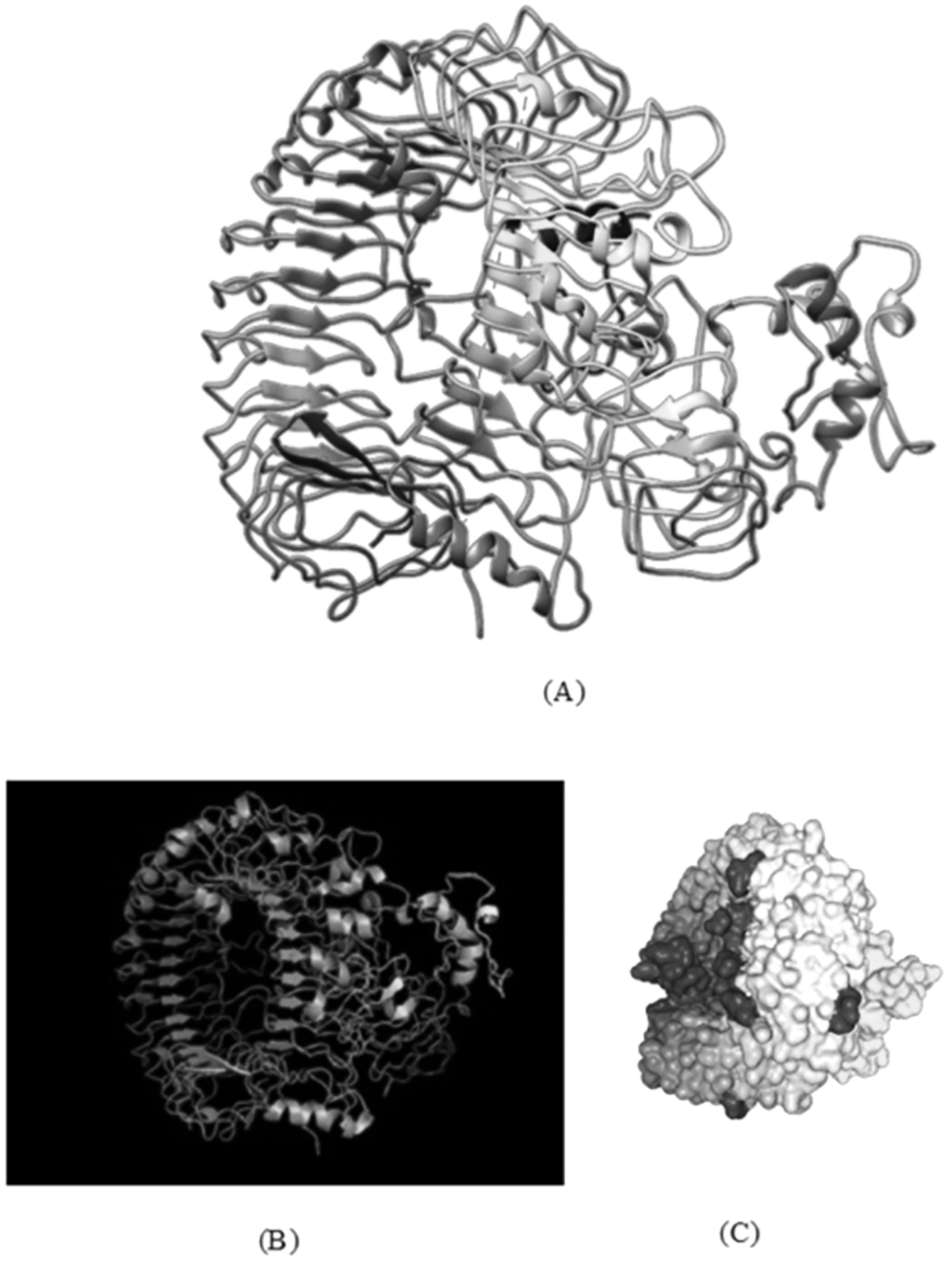
TLR-8 and vaccine construct V1 docked complex. (A) Docked complex of TLR-8 with the chimeric vaccine construct. (B) Docking complex generated *via* ClusPro server illustrating binding affinity between TLR-8 and vaccine component. The lowest energy of −1,277.5 kcal/mol was achieved for this model (complex 2). (C) Docking complex generated *via* HDOCK server which predicted the binding energy as −330.04 for protein and ligand.

With docking analysis, the binding affinity between the chimeric vaccine construct and Toll-like receptor (TLR-8) were studied. Various online tools for protein-protein docking were employed such as HDOCK, ClusPro 2.0, and the PatchDock server. The ClusPro server produced 30 protein-ligand complexes with their corresponding free binding energy as output. The lowest energy of −1,277.5 kcal/mol was obtained for the complex two that indicates spontaneous binding between the Toll-Like Receptor and the vaccine component. The HDOCK server predicted the binding energy for the protein-peptide complex as −330.04. The PatchDock generated a range of solutions, and among them, the docking assembly with the highest negative atomic contact energy (ACE) value was selected for analysis. The ACE value of the docking complex was −353.27 for solution 36 which were further evaluated for refining the complexes using FireDock, which gives the ACE value and lowest Global energy of the refined model to be 1.28 and −38.62 respectively, as obtained for solution 9 (Fig. 7).

### Molecular dynamics simulation

**Figure 8:**
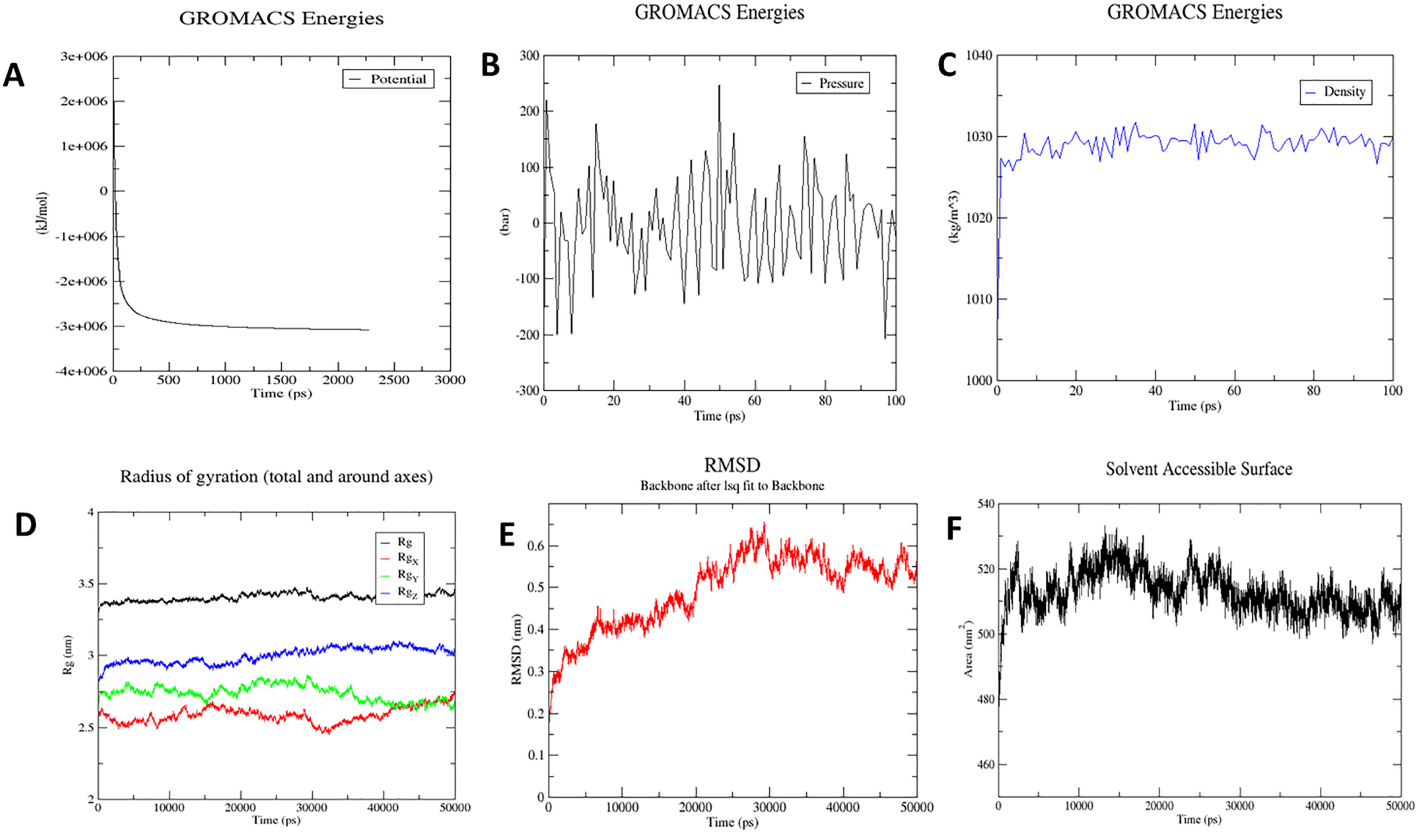
Molecular dynamics simulation study of protein-ligand complex representing. (A) Potential energy variations (B) Pressure variations plot shows that the average pressure is −2.44361 bar during 100 ps (C) Density variations; plot shows that the average density is 1,028.87 kg/m3 during 100 ps (D) Radius of gyration (E) Root mean square deviation of the docked complex backbone for the time duration of 50 ns. (F) Solvent accessible surface area of the docked complex.

The molecular dynamics simulation technique was used to investigate the three-dimensional complex structure of TLR8 and vaccine complex. An OPLS force-field was applied. The system built-in gmx solvate command was used to add 53051 number of water molecules. A total of 33 was found to be the total charge. NA^+^/Cl^−^ ions were supplied to replace the existing water molecules to neutralise the charge. The energy minimization was carried out for 50,000 steps for the steepest descent convergence, and the force was less than 1,000 Kj/mol. The average potential energy was determined to be −2,883,186.2 kJ/mol (Fig. 8A), the average temperature was determined to be 299.90 K, the average pressure was determined to be −4.29 bar after NPT equilibration (Fig. 8B), and the average density was determined to be 1,028.87 kg/m^3^ (Fig. 8C). After a 50-ns time simulation, the trajectory analysis was performed. The radius of gyration reached about 3.40 nm, signifying that the three-dimensional protein structure remained stable during MD simulation (Fig. 8D). The RMSD plot shows that the RMSD values move up to 0.49 nm and remain like that for the rest of the simulation, indicating that the complex is stable throughout time (Fig. 8E). On the other hand, SASA comes out to be 512.91 nm^2^, which denotes complex flexibility suggesting that the hydrophobic core of vaccine-TLR8 complex appeared to be exposed in aqueous surrounding (Fig. 8F).

### Characterisation of immune profile of the construct

**Figure 9:**
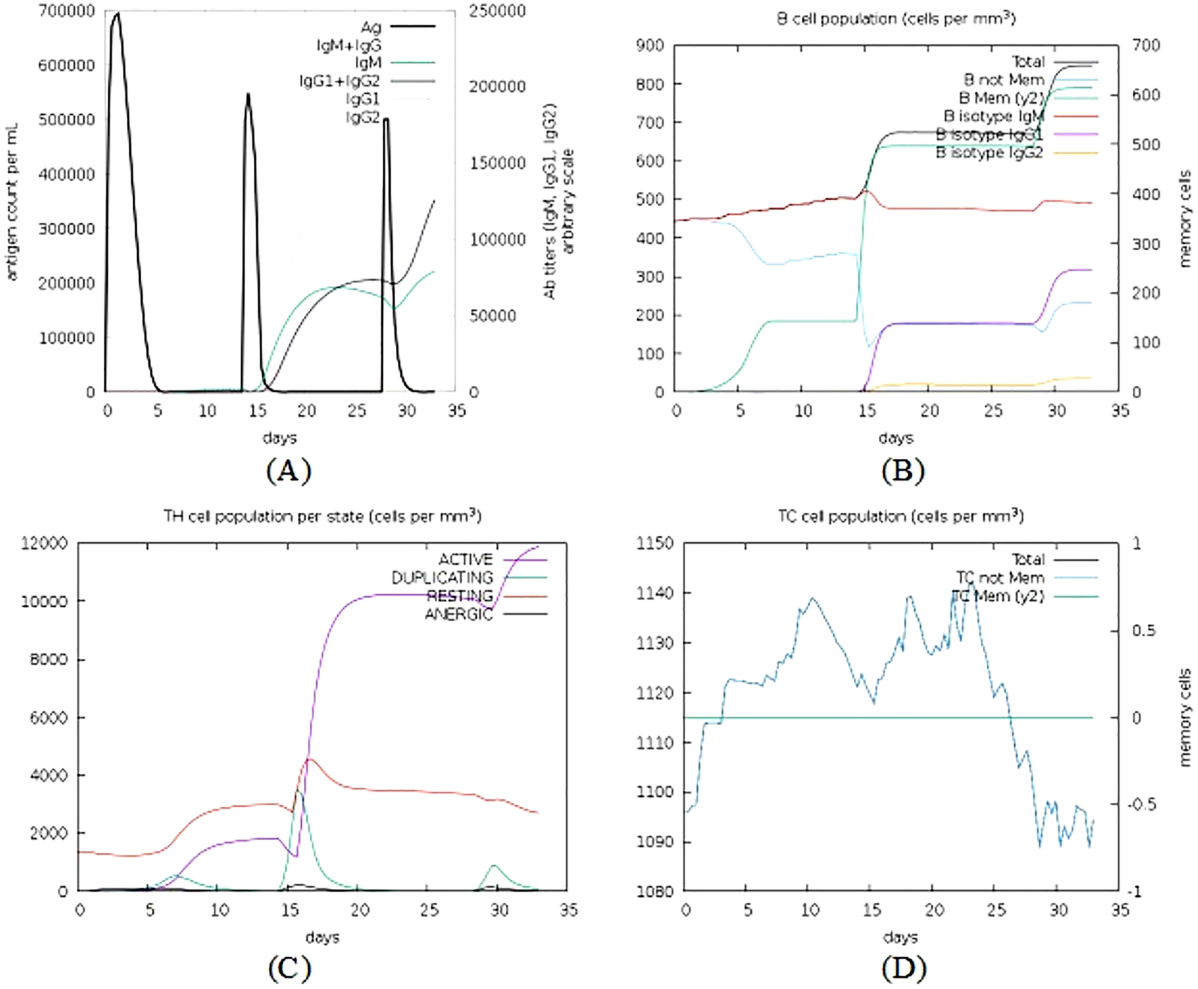
Immune simulations of the chimeric protein vaccine. (A) Production of Immunoglobulins in response to successive antigen injections (different coloured peaks corresponding to different sub-classes of immunoglobulins and antigen represented by black vertical lines). (B) Changes observed in B-cell population (C) T-helper cells per state (resting state denotes the cells not presented with antigen while anergic state denotes cells showing tolerance to antigens due to repeated exposure). (D) Changes in T-cytotoxic cell population after administration of vaccine construct V1.

The C-ImmSim simulator was used to analyse the immune response produced by the final vaccine construct. The tool generated the immune response simulations that match the response of a real immune system. Results of simulated immune responses indicate an increased surge in the induction of secondary immune responses. A B-cell population surge was observed during secondary and tertiary responses which was accompanied with rise in the levels of IgM, IgG1 + IgG2, and IgG + IgM along with the reduction in the antigen concentration (Fig. 9).

### Codon adaptation and *in silico* cloning of the chimeric protein

**Figure 10:**
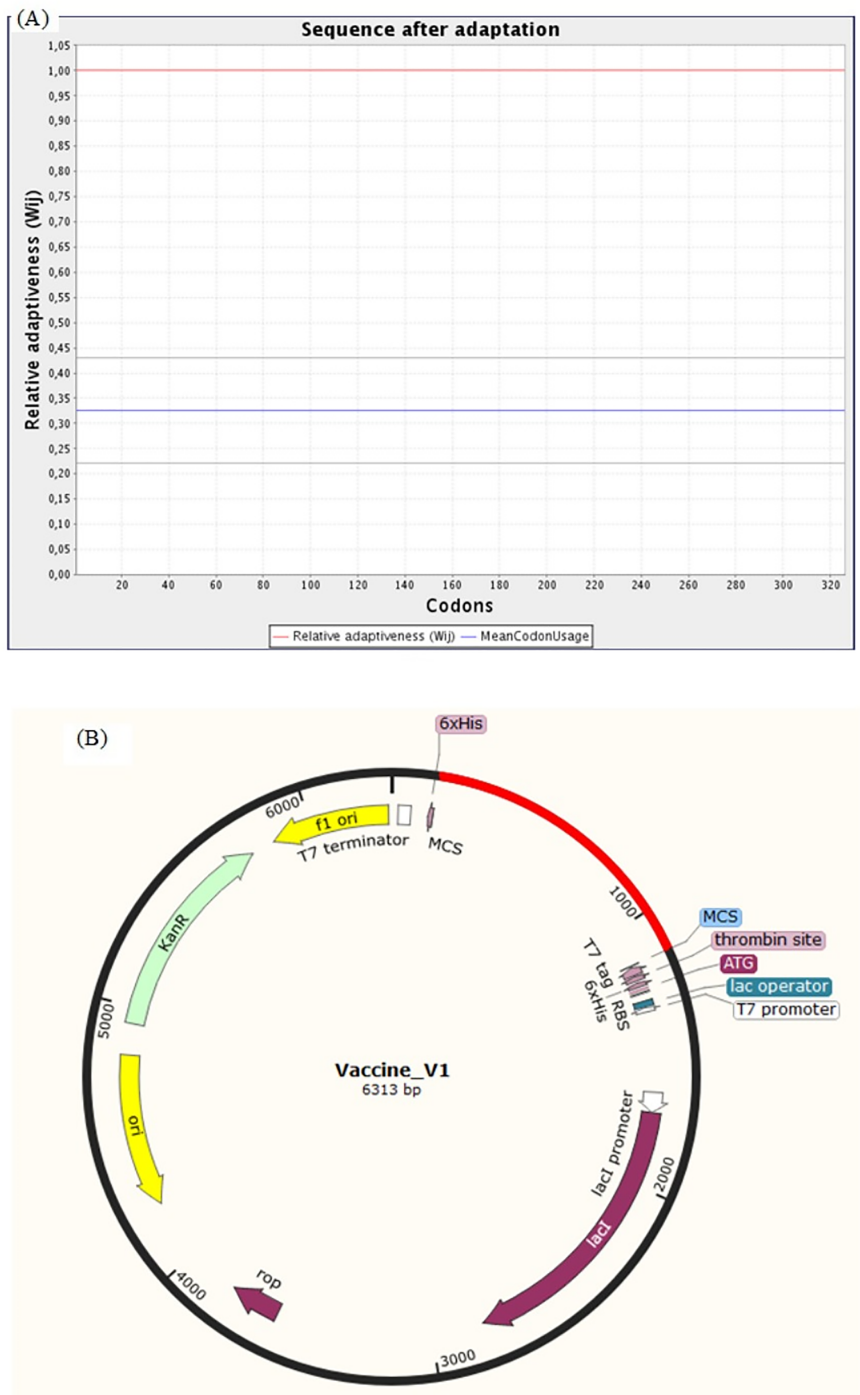
Codon adaptation and *in silico* cloning of the chimeric protein. (A) Codon adaptation result of vaccine construct V1 predicted by JCat tool predicting that the optimized codon sequence has a length of 978 nucleotides and its CAI (codon adaptation index) was predicted to be 1.0, with an average of 41.21% GC for the adapted sequence. (B) Final protein in-silico restriction cloning into pET28a (+) vector. Here, the red portion represents the gene sequence of the designed vaccine, and the black portion denotes the backbone of the vector. The DNA sequence is inserted into the MCS region of the cloning vector.

The Java Codon Adaptation Tool (JCat) was used for the optimization of codon of chimeric protein construct in *E. coli* (K12). It turned out that the optimized codon sequence has a length of 978 nucleotides and its Codon Adaptation Index (CAI) was predicted to be 1.00, with an average of 41.21% GC content (optimal range lies between 30% to 70%) for the adapted sequence. These resultant values act as determining properties indicating potentially stable expression of the constructed vaccine in the selected microbial host. For optimal gene expression, SnapGene software was employed to incorporate the adapted DNA sequence of the designed chimeric protein vaccine V1 into the *E. coli* pET-28a (+) vector by adding restriction sites which were followed by cloning of genetic sequence into the vector (Figs. 10A and 10B).

### Evaluation of vaccine construct against other SARS-CoV-2 variants

**Figure 11:**
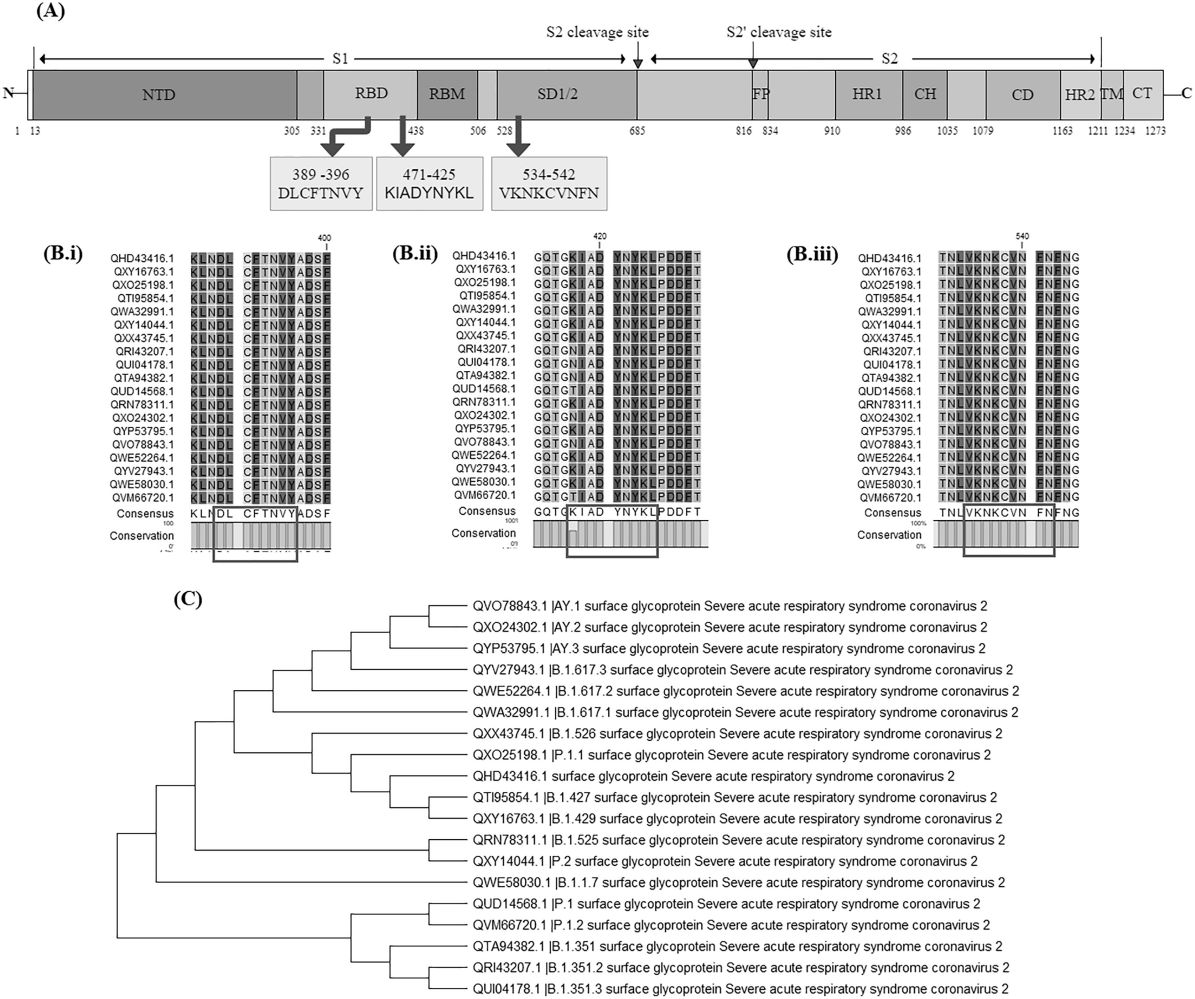
Evaluation of vaccine construct against other SARS-CoV-2 variants. (A) Schematic representation of SARS-CoV-2 Surface glycoprotein with different colours denoting different domains along with location of shortlisted BCL, HTL and CTL epitope; NTD: N-terminal domain, RBD: Receptor binding domain, RBM: Receptor binding motif, SD1/2 subdomain 1 and 2, FP: Fusion peptide, HR1: heptad repeat 1, CH: central helix, CD; connector domain, HR2: Heptad repeat 2, TM: transmembrane domain, CT: cytoplasmic tail, S1 and S2 cleavage site are protease cleavage site. DLCFTNVY: BCL epitope, KIADYNYKL: CTL epitope, VKNKCVNFN: HTL epitope. (B) Multiple sequence alignment of different SARS-CoV-2 variants done by CLC Main workbench, showing conserved epitopes (i) BCL epitope, (ii) CTL epitope and (iii) HTL epitope. (C) Phylogenetic tree of 19 SARS-CoV-2 variants. Pango lineage number has been added in the description of variants in the figure.

Surface glycoprotein sequences of 11 variants of concern {having Pango lineages: B.1.1.7, B.1.351, B.1.351.2, B.1.351.3, Delta variant (B.1.617.2, AY.1, AY.2, AY.3), P.1, P.1.1 and P.1.2} and seven variants of interest (having Pango lineages: B.1.427, B.1.429, B.1.525, B.1.526, B.1.617.1, B.1.617.3 and P.2) were extracted and aligned. The identified vaccine candidate spike protein QHD43416.1 displayed strong homology (above 99%) across these variants. The average evolutionary distance (p-distance) across all these variants was found to be 0.0063 (Supplemental Table S5). Later the conservancy analysis of the BCL, HTL and CTL epitopes used in our vaccine construct V1 was performed against 19 surface glycoprotein sequences of SARS-CoV-2 variants. It was observed that the CTL epitope conservancy ranges from 88.89−100% while the HTL and BCL epitopes were 100% conserved across all the variants (Figs. 11A, 11Bi–11Biii and 11C). This indicates that all BCL epitope: DLCFTNVY, CTL epitope: KIADYNYKL and HTL epitope: VKNKCVNFN belongs to a highly conserved domain and may prove to be an effective candidate against all these different variants (Supplemental Tables S6–S8).

### Multi-layered network of ACE2 and spike S protein

Construction of network of interacting partners of spike S proteins (Virus) and Human Interacting proteins was also performed which was used to make a multilayer network between Angiotensin Converting Enzyme 2 (ACE2) protein (Human) to study the viral host interaction (Liu et al., 2020) (Supplemental File E). Since ACE2 is a critical molecule and a potent regulator of blood pressure, body fluids and electrolyte homeostasis (Donoghue et al., 2000). Further, it was also reported that loss of ACE2 accelerates the diabetic kidney injury (Wong et al., 2007). Studies have indicated that ACE2 displays strong interaction with dipeptidyl peptidase-4 molecules. Dipeptidyl peptidase-4 (DPP4) have been shown to play a significant role in T-cell receptor (TCR)-mediated T-cell activation. Importantly, Raj et al have shown that DPP4 is an emerging functional receptor for hCoV-EMC (Raj et al., 2013). In the recent outbreak, it was reported that 60% of hospitalised patients had one or more co-existing conditions such as hypertension, cardiovascular and diabetes (Wang et al., 2020). In several studies, it has been reported that people with obesity are at a significant risk factor to suffer from complications due to COVID-19. Further, the relationship between obesity and mortality rate due to COVID-19 was investigated. We also found diseases associated with obesity such as type-2 diabetes, cardiovascular diseases and hypertension are also linked with poor prognosis in COVID-19 (Guo et al., 2020a; Kassir, 2020). We also found that several molecules implicated in obesity, diabetes, and hypertension, appear to show interactions with SARS-CoV-2 proteins as well as human proteins (ACE2 and DPP4). Thus, it might be possible to correlate the high rate of mortality of COVID-19 patients with comorbidities such as obesity, diabetes, and hypertension due to involvement of common sets of molecules. Further studies (genomic, molecular etc.) are warranted to test the hypothesis about selective disadvantage of patients suffering from metabolic syndrome X in context of coronavirus.

## Discussion

As the world is embracing a crisis, the computational community is playing an important role in fighting against the pandemic (Lu et al., 2020; Xu et al., 2020; Cleemput et al., 2020). With the recent advancements of *in-silico* based approaches and sequence-based technology; a collection of proteomic and genomic data of viral pathogens have been possible. This has made feasible the designing of peptide vaccines based on neutralizing epitopes. The immunnoinformatics strategy has been extensively studied and applied in Avian influenza A (H7N9), Monkeypox virus, Ebola virus and *Marburg marburgvirus*.

This study incorporates reverse vaccinology, bioinformatics, immunoinformatics and AI-based strategies to build a computational framework for identifying probable vaccine candidates and constructing an epitope-based vaccine against COVID-19. The framework consists of identifying surface-exposed proteins, transmembrane helices analysis, Non homology to humans, Instability analysis, antigenicity analysis, adhesion prediction and allergenicity analysis. The screening of viral proteome sequences resulted in shortlisting of the spike protein or Surface Glycoprotein of SARS-CoV-2 (accession ID QHD43416.1**)** as a potential protein target that can be used to design the vaccine. The spike protein plays an integral role in the SARS CoV-2 life cycle by cleaving into S1 glycoprotein (N-terminal) and S2 glycoprotein (C-Terminal) and exhibiting high amounts of glycosylation. S1 glycoprotein attaches the virion to the cell membrane by interacting with the host receptor, which neutralizes the antibodies in the host environment, thus causing infection. Also, S1 glycoprotein mediates the conformational changes in protein structure. The S2 glycoprotein is used in mediating the fusion of virion and cell membranes by enacting the role of class 1 viral fusion protein.

The shortlisted protein was subjected to computation of various physicochemical properties like number of amino acids, GRAVY value, extinction coefficient, molecular weight, instability index, theoretical pI, aliphatic index and cysteine disulfide bond score. Tools namely ProPred, ProPred-I and BcePred were employed for the determination of all the possible epitopes for T cells and B cells. B and T lymphocytic cells play an important role in developing acquired immunity. The antigens, after being recognised by APC (Antigen Presenting Cells) are presented *via* MHC-II molecule to helper-T cells which further activates B-cells. The B-cells produce antibodies whereas T-helper cells also activate macrophages and cytotoxic T-lymphocytes. All these epitopes were found in Receptor Binding Domain or SD1/2 domain that are highly conserved (RBD) within SARS-CoV-2 S protein. In a study by Ong et al. (2020), the authors investigated entire proteome, including the S protein and five non-structural proteins (nsp3, 3CL-pro, and nsp8-10) and labelled them as adhesins, which are crucial to the viral adhering and host invasion. They also found nsp3 to be more conserved among SARS-CoV-2, SARS-CoV, and MERS-CoV than among 15 coronaviruses infecting human and other animals. The protein was also predicted to contain promiscuous MHC-I and MHC-II T-cell epitopes, and linear B-cell epitopes localized in specific locations and functional domains of the protein. They also used a pipeline called Vaxign-ML for target predictions.

Using immunoinformatic and docking studies, Bhattacharya et al. (2020) identified potential epitopes and docking complexes of constructed vaccines and TLR5. Another group of scientists have also identified a set of B-cell and T-cell epitopes derived from the spike (S) and nucleocapsid (N) proteins that map identically to SARS-CoV-2 proteins under the assumption that no mutation was seen in limited dataset of 120 available SARS-CoV-2 sequences (as of 21 February 2020). This assumption of zero mutation rate has changed in the light of new data submitted since February 2020 (Ahmed, Quadeer & McKay, 2020). Robson (2020) reported a specific sequence motif “KRSFIEDLLFNKV” as a conserved and interesting target. He also reported that this region is associated closely with known cleavage sites of the SARS virus that are believed to be required for virus activation for cell entry (Robson, 2020). In another study, Grifoni et al. (2020) used bioinformatics approaches to identify *a priori* potential B and T cell epitopes for SARS-CoV-2 using IEDB resources. They also described immune-dominant regions located in the S1 subunit in the CTD2 and CTD3 (C-terminal domain), and in the HR1 domain of the S2 subunit. Kiyotani et al. (2020) comprehensively screened potential SARS-CoV-2-derived, HLA-class I and II-presented epitopes for 43 *HLA* alleles that are common in the Japanese population, and identified 2013 and 1,399 epitopes, respectively. They found that 781 HLA-class I and 418 HLA-class II epitopes were common between SARS-CoV-2 and SARS-CoV. Researchers have tested 15 epitope-HLA-binding prediction tools, and using an *in vitro* peptide MHC stability assay, and assessed 777 peptides that were predicted to be good binders across 11 MHC allotypes (Prachar et al., 2020). A research group recently found a cross-protective epitope between the spike proteins of SARS-CoV-2 and SARS-CoV, and successfully found the cross-protective epitopes in the RBDs of the spike proteins (Qiu et al., 2020). Further, another study found that the spike RBD of SARS-CoV-2 bound potently to angiotensin-converting enzyme 2 (ACE2), the host cell receptor of SARS-CoV (Tian et al., 2020).

Studies indicate that HLA variations are associated with susceptibility or resistance to malaria, tuberculosis, leprosy, HIV, and hepatitis virus persistence (Blackwell, Fakiola & Castellucci, 2020). A report also suggests that human coronavirus OC43 interacts with HLA class I molecules at the cell surface to establish infection (Collins, 1994). Further, one study (Lin et al., 2003) indicates the association of HLA-B* 4601 with the severity of SARS infection in Asian population. In our work, we employed computational strategies (*i.e*., molecular docking) to check interaction of viral peptides with the commonly found human allele (HLA*B7) and Wuhan region (HLA*A2). Additionally, the stability of vaccine-TLR8 complex was further studied by molecular docking and simulation studies. Furthermore, *in vitro* and *in vivo* studies should be conducted to confirm the safety and potency of the predicted vaccine candidates. We suggest further wet lab-based studies and procedures, using animal models for experimental validation of our predicted vaccine candidates.

## Conclusion

In this study, the whole proteome of SARS-CoV-2 was screened using reverse vaccinology, bioinformatics and immunoinformatic approaches to identify potential vaccine candidates. Through our investigation, we arrive at a conclusion that the spike glycoprotein is one of the major protein responsible for pathophysiology of SARS-CoV-2. The potential epitopes were identified through a robust process and employed for vaccine construction, using which, several potential vaccine constructs were obtained. Therefore, our study will ease the development of appropriate therapeutic and prompt the future vaccine development against COVID-19 and this could serve an important milestone in developing an antiviral vaccine against SARS-CoV-2.

## Supplemental Information

10.7717/peerj.13380/supp-1 Supplemental Information 1 Supplementary Table (S1-S4).

10.7717/peerj.13380/supp-2 Supplemental Information 2 Estimates of Evolutionary Divergence between Sequences of surface glycoprotein (Spike protein) belonging to different variants of SARS-CoV-2.

10.7717/peerj.13380/supp-3 Supplemental Information 3 Conservancy analysis of B-cell epitopes across 19 SARS-CoV-2 with variants of concerns.

10.7717/peerj.13380/supp-4 Supplemental Information 4 Conservancy analysis of CTL epitope against 19 SARS-COV-2 including variants of concerns.

10.7717/peerj.13380/supp-5 Supplemental Information 5 Conservancy analysis of HTL epitope across 19 SARS-CoV-2 variants including variants of concerns.

10.7717/peerj.13380/supp-6 Supplemental Information 6 Supplementary Files encompassing A to E.

10.7717/peerj.13380/supp-7 Supplemental Information 7 Comparison of shortlisted epitopes with epitopes predicted by other tools.

10.7717/peerj.13380/supp-8 Supplemental Information 8 Prediction of BCL epitopes using various *in silico* tools and resources.

10.7717/peerj.13380/supp-9 Supplemental Information 9 Prediction of CTL epitopes using various *in silico* tools and resources.

10.7717/peerj.13380/supp-10 Supplemental Information 10 Prediction of HTL epitopes using various *in silico* tools and resources.

## Additional Information and Declarations

### Data Availability

The following information was supplied regarding data availability: The raw data is available in the Supplemental Files.
