# Supplementary material for: Identification of vaccine targets & design of vaccine against SARS-CoV-2 coronavirus using computational and deep learning-based approaches
Source: PeerJ. 2022 May 19;10:e13380. doi: 10.7717/peerj.13380 (PMC9124463; doi:10.7717/peerj.13380)
Supplement: Supplemental Information 1 [file peerj-10-13380-s001.docx]

**Supplementary Table S1**: MHC class-I allele binding epitopes predicted using Propred-I with their antigenicity scores.

| **Sl. No** | **Peptide** | **Position** | **Score** | **Binding Allele** |
| --- | --- | --- | --- | --- |
| 1 | KIADYNYKL | 417 | 1.66 | HLA-A2, HLA-A*02:01, HLA-A*02:05, HLA-A*11:01, HLA-A24, HLA-A3, HLA-A*31:01, HLA-B*27:05, HLA-B*35:01, HLA-B*38:01, HLA-B*39:01, HLA-B*39:02, HLA-B*07:02, HLA-Cw*04:01, HLA-Cw*07:02, MHC-Dd, |
| 2 | VVVLSFELL | 510 | 1.09 | HLA-A*02:05, HLA-A*11:01, HLA-A24, HLA-A3, HLA-A*31:01, HLA-A*33:02, HLA-A68.1, HLA-B*37:01, HLA-B*39:01, HLA-B*53:01, HLA-B*51, HLA-B7, HLA-Cw*03:01, MHC-Db, MHC-Db revised, MHC-Kb, MHC-Kd |
| 3 | TLDSKTQSL | 109 | 1.06 | HLA-A1,HLA-A2,HLA-A*0201, HLA-A3,HLA-A*3101,HLA-A*3302, HLA-A20 Cattle, HLA-A2.1, HLA-B*2702,HLA-B*2705, HLA-B*3801,HLA-B*3901,HLA-B8, HLA-Cw*0401,HLA-Cw*0602, MHC-Dd, |
| 4. | GKQGNFKNL | 181 | 1.06 | HLA-A2,HLA-A20Cattle,HLA-B*3902, HLA-Cw*0301,MHC-Db,MHC-Dbrevised,MHC-Dd, MHC-Kb, |
| 5. | VRDLPQGFS | 213 | 1.05 | HLA-B*2702,  HLA-B*2705 |
| 6 | PWYIWLGFI | 1213 | 1.03 | HLA-A2.1, HLA-B*5401 |
| 7 | NFGAISSVL | 969 | 0.98 | HLA-A24, HLA-B*3801, HLA-B*3902, HLA-B*5201,  HLA-Cw*0401, HLA-Cw*0602, HLA-Cw*0702, MHC-Kd |
| 8 | QGFSALEPL | 218 | 0.84 | HLA-B14,  HLA-B*3901, HLA-B40, HLA-B*5101 , HLA-B*5102, HLA-B*5103, HLA-B*5201, HLA-B*5401,  HLA-B61, HLA-B7, HLA-B*0702, HLA-Cw*0401, HLA-Cw*0702, MHC-Dd, MHC-Kb, MHC-Kd |

**Supplementary Table S2**: Physicochemical profiling of selected peptides by ToxinPred tool

| **Sl. No** | [**Peptide Sequence**](https://webs.iiitd.edu.in/raghava/toxinpred/multi_submitfreq_S.php?ran=15951) | [**SVM Score**](https://webs.iiitd.edu.in/raghava/toxinpred/multi_submitfreq_S.php?ran=15951) | [**Prediction**](https://webs.iiitd.edu.in/raghava/toxinpred/multi_submitfreq_S.php?ran=15951) | [**Hydrophobicity**](https://webs.iiitd.edu.in/raghava/toxinpred/multi_submitfreq_S.php?ran=15951) | [**Hydropathicity**](https://webs.iiitd.edu.in/raghava/toxinpred/multi_submitfreq_S.php?ran=15951) | [**Hydrophilicity**](https://webs.iiitd.edu.in/raghava/toxinpred/multi_submitfreq_S.php?ran=15951) | [**Charge**](https://webs.iiitd.edu.in/raghava/toxinpred/multi_submitfreq_S.php?ran=15951) | [**Mol wt**](https://webs.iiitd.edu.in/raghava/toxinpred/multi_submitfreq_S.php?ran=15951) |
| --- | --- | --- | --- | --- | --- | --- | --- | --- |
| 1 | KIADYNYKL | -0.59 | Non-Toxin | -0.22 | -0.81 | 0.06 | 1 | 1127.43 |
| 2 | VVVLSFELL | -1.29 | Non-Toxin | 0.33 | 2.5 | -1.01 | -1 | 1018.4 |
| 3 | TLDSKTQSL | -1.34 | Non-Toxin | -0.26 | -0.7 | 0.27 | 0 | 992.22 |
| 4 | GKQGNFKNL | -1.12 | Non-Toxin | -0.3 | -1.39 | 0.26 | 2 | 1005.28 |
| 5 | VRDLPQGFS | -1.17 | Non-Toxin | -0.18 | -0.39 | 0.08 | 0 | 1018.26 |
| 6 | PWYIWLGFI | -0.3 | Non-Toxin | 0.38 | 1.17 | -1.89 | 0 | 1194.58 |
| 7 | NFGAISSVL | -0.91 | Non-Toxin | 0.18 | 1.29 | -0.81 | 0 | 907.16 |
| 8 | QGFSALEPL | -1.03 | Non-Toxin | 0.05 | 0.27 | -0.34 | -1 | 961.21 |

**Supplementary Table S3:** MHC class-II allele binding epitopes predicted using Propred with their antigenicity scores

| **Sl.No.** | **Sequence** | **Position** | **Score** | **Binding Allele** |
| --- | --- | --- | --- | --- |
| 1. | VKNKCVNFN | 533 | 2.05 | ['DRB1_0306', 'DRB1_0307', 'DRB1_0308', 'DRB1_0311', 'DRB1_0402', 'DRB1_0405', 'DRB1_0410', 'DRB1_0801', 'DRB1_0802', 'DRB1_0804', 'DRB1_0806', 'DRB1_0813', 'DRB1_0817', 'DRB1_1102', 'DRB1_1114', 'DRB1_1120', 'DRB1_1121', 'DRB1_1301', 'DRB1_1302', 'DRB1_1304', 'DRB1_1321', 'DRB1_1322', 'DRB1_1323', 'DRB1_1327', 'DRB1_1328'] |
| 2. | YRFNGIGVT | 903 | 1.76 | ['DRB1_0101', 'DRB1_0102', 'DRB1_0301', 'DRB1_0305', 'DRB1_0306', 'DRB1_0307', 'DRB1_0308', 'DRB1_0309', 'DRB1_0311', 'DRB1_0401', 'DRB1_0402', 'DRB1_0404', 'DRB1_0405', 'DRB1_0408', 'DRB1_0410', 'DRB1_0421', 'DRB1_0423', 'DRB1_0426', 'DRB1_0701', 'DRB1_0703', 'DRB1_0801', 'DRB1_0802', 'DRB1_0804', 'DRB1_0806', 'DRB1_0813', 'DRB1_0817', 'DRB1_1101', 'DRB1_1102', 'DRB1_1104', 'DRB1_1106', 'DRB1_1107', 'DRB1_1114', 'DRB1_1120', 'DRB1_1121', 'DRB1_1128', 'DRB1_1301', 'DRB1_1302', 'DRB1_1304', 'DRB1_1305', 'DRB1_1307', 'DRB1_1311', 'DRB1_1321', 'DRB1_1322', 'DRB1_1323', 'DRB1_1327', 'DRB1_1328', 'DRB1_1501', 'DRB1_1502', 'DRB1_1506', 'DRB5_0101', 'DRB5_0105'] |
| 3. | VVFLHVTYV | 1059 | 1.51 | ['DRB1_0101', 'DRB1_0102', 'DRB1_0301', 'DRB1_0305', 'DRB1_0306', 'DRB1_0307', 'DRB1_0308', 'DRB1_0309', 'DRB1_0311', 'DRB1_0401', 'DRB1_0402', 'DRB1_0404', 'DRB1_0405', 'DRB1_0408', 'DRB1_0410', 'DRB1_0421', 'DRB1_0423', 'DRB1_0426', 'DRB1_0701', 'DRB1_0703', 'DRB1_0801', 'DRB1_0802', 'DRB1_0804', 'DRB1_0806', 'DRB1_0813', 'DRB1_0817', 'DRB1_1101', 'DRB1_1102', 'DRB1_1104', 'DRB1_1106', 'DRB1_1107', 'DRB1_1114', 'DRB1_1120', 'DRB1_1121', 'DRB1_1128', 'DRB1_1301', 'DRB1_1302', 'DRB1_1304', 'DRB1_1305', 'DRB1_1307', 'DRB1_1311', 'DRB1_1321', 'DRB1_1322', 'DRB1_1323', 'DRB1_1327', 'DRB1_1328', 'DRB1_1501', 'DRB1_1502', 'DRB1_1506', 'DRB5_0101', 'DRB5_0105'] |
| 4. | FKCYGVSPT | 376 | 1.51 | ['DRB1_0405', 'DRB1_0408', 'DRB1_0801', 'DRB1_0802', 'DRB1_0804', 'DRB1_0806', 'DRB1_0813', 'DRB1_0817', 'DRB1_1114', 'DRB1_1120', 'DRB1_1302', 'DRB1_1307', 'DRB1_1323', 'DRB1_1501', 'DRB1_1502', 'DRB1_1506'] |
| 5. | VNLTTRTQL | 15 | 1.34 | ['DRB1_0102', 'DRB1_0301', 'DRB1_0306', 'DRB1_0307', 'DRB1_0308', 'DRB1_0311', 'DRB1_0701', 'DRB1_0703', 'DRB1_0813', 'DRB1_1107', 'DRB1_1501', 'DRB1_1502', 'DRB1_1506', 'DRB5_0101', 'DRB5_0105'] |
| 6. | IGINITRFQ | 230 | 1.33 | ['DRB1_0102', 'DRB1_0301', 'DRB1_0306', 'DRB1_0307', 'DRB1_0308', 'DRB1_0311', 'DRB1_0701', 'DRB1_0703', 'DRB1_0813', 'DRB1_1107', 'DRB1_1501', 'DRB1_1502', 'DRB1_1506', 'DRB5_0101', 'DRB5_0105'] |
| 7. | LVKNKCVNF | 532 | 1.32 | ['DRB1_0101', 'DRB1_0102', 'DRB1_0301', 'DRB1_0305', 'DRB1_0306', 'DRB1_0307', 'DRB1_0308', 'DRB1_0309', 'DRB1_0311', 'DRB1_0401', 'DRB1_0402', 'DRB1_0410', 'DRB1_0421', 'DRB1_0426', 'DRB1_0701', 'DRB1_0703', 'DRB1_0806', 'DRB1_1102', 'DRB1_1104', 'DRB1_1106', 'DRB1_1107', 'DRB1_1114', 'DRB1_1120', 'DRB1_1121', 'DRB1_1128', 'DRB1_1301', 'DRB1_1302', 'DRB1_1304', 'DRB1_1305', 'DRB1_1311', 'DRB1_1322', 'DRB1_1323', 'DRB1_1327', 'DRB1_1328', 'DRB1_1501', 'DRB1_1506'] |
| 8. | VVIGIVNNT | 1127 | 1.3 | ['DRB1_0101', 'DRB1_0102', 'DRB1_0301', 'DRB1_0305', 'DRB1_0306', 'DRB1_0307', 'DRB1_0308', 'DRB1_0309', 'DRB1_0311', 'DRB1_0401', 'DRB1_0402', 'DRB1_0404', 'DRB1_0405', 'DRB1_0408', 'DRB1_0410', 'DRB1_0421', 'DRB1_0423', 'DRB1_0426', 'DRB1_0701', 'DRB1_0703', 'DRB1_0801', 'DRB1_0802', 'DRB1_0804', 'DRB1_0806', 'DRB1_0813', 'DRB1_0817', 'DRB1_1101', 'DRB1_1102', 'DRB1_1104', 'DRB1_1106', 'DRB1_1107', 'DRB1_1114', 'DRB1_1120', 'DRB1_1121', 'DRB1_1128', 'DRB1_1301', 'DRB1_1302', 'DRB1_1304', 'DRB1_1305', 'DRB1_1307', 'DRB1_1311', 'DRB1_1321', 'DRB1_1322', 'DRB1_1323', 'DRB1_1327', 'DRB1_1328', 'DRB1_1501', 'DRB1_1502', 'DRB1_1506'] |

**Supplementary Table S4**: Physicochemical properties of selected MHC-II peptides

| **Sl. No** | [**Peptide Sequence**](https://webs.iiitd.edu.in/raghava/toxinpred/multi_submitfreq_S.php?ran=15951) | [**SVM Score**](https://webs.iiitd.edu.in/raghava/toxinpred/multi_submitfreq_S.php?ran=15951) | [**Prediction**](https://webs.iiitd.edu.in/raghava/toxinpred/multi_submitfreq_S.php?ran=15951) | [**Hydrophobicity**](https://webs.iiitd.edu.in/raghava/toxinpred/multi_submitfreq_S.php?ran=15951) |
| --- | --- | --- | --- | --- |
| 1. | VKNKCVNFN | -0.34 | Non-Toxin | -0.27 |
| 2. | YRFNGIGVT | -1.3 | Non-Toxin | -0.04 |
| 3. | VVFLHVTYV | -1.06 | Non-Toxin | 0.3 |
| 4. | FKCYGVSPT | -0.25 | Non-Toxin | -0.03 |
| 5. | VNLTTRTQL | -1.09 | Non-Toxin | -0.23 |
| 6. | IGINITRFQ | -1.02 | Non-Toxin | -0.03 |
| 7. | LVKNKCVNF | -0.27 | Non-Toxin | -0.14 |
| 8. | VVIGIVNNT | -0.98 | Non-Toxin | -0.2 |
