# Supplementary material for: Identification of vaccine targets & design of vaccine against SARS-CoV-2 coronavirus using computational and deep learning-based approaches
Source: PeerJ. 2022 May 19;10:e13380. doi: 10.7717/peerj.13380 (PMC9124463; doi:10.7717/peerj.13380)
Supplement: Supplemental Information 6 [file peerj-10-13380-s006.docx]

**Identification of vaccine targets & design of vaccine against SARS-CoV-2 coronavirus using computational and deep learning-based approaches.**

**Bilal Ahmed Abbasi ^1*^, Devansh Saraf ^1^, Trapti Sharma ^1^, Robin Sinha ^1^, Shachee Singh ^1^, Shriya Sood^1^, Pranjay Gupta^1^, Akshat Gupta^1^, Kartik Mishra^1^, Priya Kumari^1^, Kamal Rawal ^# 1, *^**

1. Center for Computational Biology and Bioinformatics, Amity Institute of Biotechnology, Amity University Uttar Pradesh, India.

**#Corresponding Author**

*** Equal Contribution**

Email Id: kamal.rawal@gmail.com

Center for Computational Biology and Bioinformatics, AIB

Amity University, Noida.

**Supplementary File A**

**Strategy of vaccine construction:**

**Strategy 1:**

8 repeats of highest scoring epitope each of MHC-I, MHC-II and B-cell epitopes were linked to adjuvant and each other via linkers. Three vaccine V1, V2 and V3 were constructed using this approach. The vaccine sequence in all these three were same, however, each has different adjuvant namely, β defensin, L7/L12 50s ribosomal protein and HABA protein, respectively.

**Strategy 2:**

Top scoring 8 epitopes each of MHC-I, MHC-II and B-cell epitopes were linked to adjuvant via appropriate linkers. Vaccine constructs V4, V5 and V6 were generated following this approach and β defensin, 50s L7/L12 ribosomal protein and HABA protein, respectively were used as adjuvants.

**Linkers Used:**

1. EAAAK linker for joining adjuvant with MHC class-I (or CTL) epitope.

2. GGGS linker for linking CTL epitopes with among themselves.

3. AAY linker for joining CTL epitope with MHC-II (or HTL) epitopes and HTL epitopes among themselves

4. KK linker for linking HTL epitope with B-cell epitope and B-cell epitopes among themselves.

**Table A:** Protein sequence of the vaccine constructs produced by following the mentioned strategies. The colour coding is as follows: Adjuvant (Green), Linkers (Blue), MHC class-I epitopes or CTL epitopes (Violet), MHC class-II or HTL epitopes (Red) and B- cell epitopes (Black).

| **Vaccine Construct** | **Vaccine sequence** |
| --- | --- |
| V1  With β defensin as adjuvant | GIINTLQKYYCRVRGGRCAVLSCLPKEEQIGKCSTRGRKCCRRKKEAAAKKIADYNYKLGGGSKIADYNYKLGGGSKIADYNYKLGGGSKIADYNYKLGGGSKIADYNYKLGGGSKIADYNYKLGGGSKIADYNYKLGGGSKIADYNYKLAAYVKNKCVNFNAAYVKNKCVNFNAAYVKNKCVNFNAAYVKNKCVNFNAAYVKNKCVNFNAAYVKNKCVNFNAAYVKNKCVNFNAAYVKNKCVNFNKKDLCFTNVYKKDLCFTNVYKKDLCFTNVYKKDLCFTNVYKKDLCFTNVYKKDLCFTNVYKKDLCFTNVYKKDLCFTNVY |
| V2  With L7/L12 50s ribosomal protein | MSDINKLAETLVNLKIVEVNDLAKILKEKYGLDPSANLAIPSLPKAEILDKSKEKTSFDLILKGAGSAKLTVVKRIKDLIGLGLKESKDLVDNVPKHLKKGLSKEEAESLKKQLEEVGAEVELKEAAAKKIADYNYKLGGGSKIADYNYKLGGGSKIADYNYKLGGGSKIADYNYKLGGGSKIADYNYKLGGGSKIADYNYKLGGGSKIADYNYKLGGGSKIADYNYKLAAYVKNKCVNFNAAYVKNKCVNFNAAYVKNKCVNFNAAYVKNKCVNFNAAYVKNKCVNFNAAYVKNKCVNFNAAYVKNKCVNFNAAYVKNKCVNFNKKDLCFTNVYKKDLCFTNVYKKDLCFTNVYKKDLCFTNVYKKDLCFTNVYKKDLCFTNVYKKDLCFTNVYKKDLCFTNVY |
| V3  With HABA protein | MAENPNIDDLPAPLLAALGAADLALATVNDLIANLRERAEETRAETRTRVEERRARLTKFQEDLPEQFIELRDKFTTEELRKAAEGYLEAATNRYNELVERGEAALQRLRSQTAFEDASARAEGYVDQAVELTQEALGTVASQTRAVGERAAKLVGIELEAAAKKIADYNYKLGGGSKIADYNYKLGGGSKIADYNYKLGGGSKIADYNYKLGGGSKIADYNYKLGGGSKIADYNYKLGGGSKIADYNYKLGGGSKIADYNYKLAAYVKNKCVNFNAAYVKNKCVNFNAAYVKNKCVNFNAAYVKNKCVNFNAAYVKNKCVNFNAAYVKNKCVNFNAAYVKNKCVNFNAAYVKNKCVNFNKKDLCFTNVYKKDLCFTNVYKKDLCFTNVYKKDLCFTNVYKKDLCFTNVYKKDLCFTNVYKKDLCFTNVYKKDLCFTNVY |
| V4  With β defensin as adjuvant | GIINTLQKYYCRVRGGRCAVLSCLPKEEQIGKCSTRGRKCCRRKKEAAAKKIADYNYKLGGGSVVVLSFELLGGGSTLDSKTQSLGGGS GKQGNFKNLGGGSVRDLPQGFSGGGSPWYIWLGFIGGGSNFGAISSVLGGGSQGFSALEPLAAYVKNKCVNFNAAYYRFNGIGVTAAYVVFLHVTYVAAYFKCYGVSPTAAYVNLTTRTQLAAYIGINITRFQAAYLVKNKCVNFAAYVVIGIVNNTKKDLCFTNVYKKYYVGYLQPRKKEPVLKGVKLHYTKKLIDLQELKKTEILPVSKKEILDITPCSFGGVSVITPGKKSVVNIQKKKYQPYRVVVLSFELLH |
| V5  With L7/L12 50s ribosomal protein | MSDINKLAETLVNLKIVEVNDLAKILKEKYGLDPSANLAIPSLPKAEILDKSKEKTSFDLILKGAGSAKLTVVKRIKDLIGLGLKESKDLVDNVPKHLKKGLSKEEAESLKKQLEEVGAEVELKEAAAKKIADYNYKLGGGSVVVLSFELLGGGSTLDSKTQSLGGGSGKQGNFKNLGGGSVRDLPQGFSGGGSPWYIWLGFIGGGSNFGAISSVLGGGSQGFSALEPLAAYVKNKCVNFNAAYYRFNGIGVTAAYVVFLHVTYVAAYFKCYGVSPTAAYVNLTTRTQLAAYIGINITRFQAAYLVKNKCVNFAAYVVIGIVNNTKKDLCFTNVYKKYYVGYLQPRKKEPVLKGVKLHYTKKLIDLQELKKTEILPVSKKEILDITPCSFGGVSVITPGKKSVVNIQKKKYQPYRVVVL SFELLH |
| V6  With HABA protein | MAENPNIDDLPAPLLAALGAADLALATVNDLIANLRERAEETRAETRTRVEERRARLTKFQEDLPEQFIELRDKFTTEELRKAAEGYLEAATNRYNELVERGEAALQRLRSQTAFEDASARAEGYVDQAVELTQEALGTVASQTRAVGERAAKLVGIELEAAAKKIADYNYKLGGGSVVVLSFELLGGGSTLDSKTQSLGGGSGKQGNFKNLGGGSVRDLPQGFSGGGSPWYIWLGFIGGGSNFGAISSVLGGGSQGFSALEPLAAYVKNKCVNFNAAYYRFNGIGVTAAYVVFLHVTYVAAYFKCYGVSPTAAYVNLTTRTQLAAYIGINITRFQAAYLVKNKCVNFAAYVVIGIVNNTKKDLCFTNVYKKYYVGYLQPRKKEPVLKGVKLHYTKKLIDLQELKKTEILPVSKKEILDITPCSFGGVSVITPGKKSVVNIQKKKYQPYRVVVL SFELLH |

**Supplementary File B**

**Antigenicity, Allergenicity, Solubility and Physicochemical analysis of vaccine constructs V2, V3, V4, V5 and V6.**

Each vaccine construct was analysed through various tools to evaluate their comparative antigenicity, allergenicity, solubility and other physicochemical properties using VaxiJen v2.0 server, Algpred, Protein-Sol and Protparam respectively.

**Table B:** Tabular representation showing the antigenicity, allergenicity, solubility and various physicochemical properties of vaccine constructs V2-V6. Antigenicity was predicted by VaxiJen having threshold 0.4. Allergenicity was evaluated by Algpred server. Solubility was predicted by Protein-Sol tool which has a threshold value of 0.45 (value > 0.45 indicates higher solubility than average soluble E. coli protein from experimental dataset utilised by this tool). Other physicochemical properties were predicted by Protparam server including Molecular weight, Number of amino acids, Theoretical isoelectric point pI, half-life, Instability index (II) (II <40 indicates stability), Aliphatic index and Hydropathicity (GRAVY value).

| **Properties** | **Vaccine Construct** | | | | |
| --- | --- | --- | --- | --- | --- |
|  | **V2** | **V3** | **V4** | **V5** | **V6** |
| **Antigenicity** | 1.03 | 0.98 | 0.9016 | 0.8272 | 0.7899 |
| **Allergenicity** | Non- Allergen | Non- Allergen | Non- Allergen | Non- Allergen | Non- Allergen |
| **Solubility** | 0.659  (higher solubility than average soluble E. coli protein) | 0.638  (higher solubility than average soluble E. coli protein) | 0.636  (higher solubility than average soluble E. coli protein) | 0.508  (higher solubility than average soluble E. coli protein) | 0.423  (lesser solubility than average soluble E. coli protein) |
| **Molecular weight (kDa)** | 45.23 | 49.30 | 37.66 | 46.06 | 50.13 |
| **Number of amino acids** | 405 | 440 | 346 | 425 | 460 |
| **Theoretical pI** | 9.41 | 9.17 | 9.79 | 9.54 | 9.20 |
| **Half- life** | 30 hrs in mammalian reticulocytes in vitro, and >20 hours in yeast and >10 hours in *E. coli* in vivo | 30 hrs in mammalian reticulocytes in vitro, and >20 hours in yeast and >10 hours in *E. coli* in vivo | 30 hrs in mammalian reticulocytes in vitro, and >20 hours in yeast and >10 hours in *E. coli* in vivo | 30 hrs in mammalian reticulocytes in vitro, and >20 hours in yeast and >10 hours in *E. coli* in vivo | 30 hrs in mammalian reticulocytes in vitro, and >20 hours in yeast and >10 hours in *E. coli* in vivo |
| **Instability index** | 10.01 | 17.60 | 31.65 | 27.82 | 33.72 |
| **Aliphatic index** | 83.56 | 76.14 | 90.66 | 101.58 | 93.11 |
| **Hydropathicity**  **(GRAVY value)** | -0.380 | -0.454 | -0.025 | -0.25 | -0.122 |

**Supplementary File C**

**Secondary structure prediction of vaccine constructs V2, V3, V4, V5, V6**

Secondary structure of all the constructed vaccine sequences was predicted via PSIPRED server its secondary structure composition via CFSSP server.

**Vaccine construct V2:**


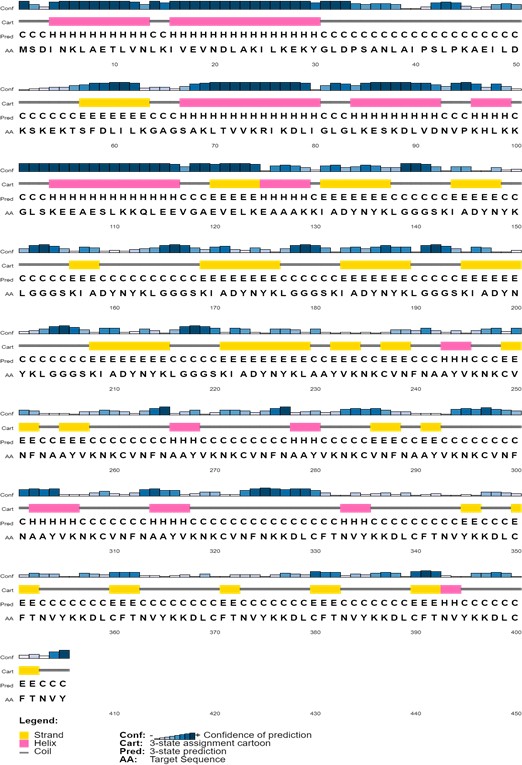


Figure C (i): Graphical representation of secondary structure features of the proposed subunit vaccine sequence construct V2.

**Vaccine construct V3:**


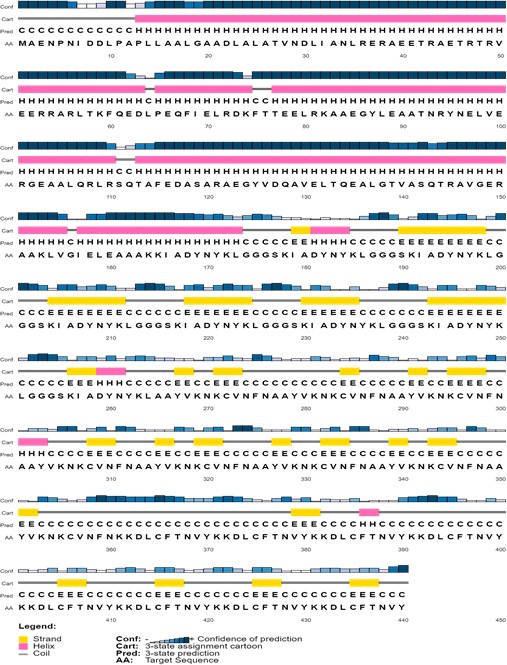


Figure C(ii): Graphical representation of secondary structure features of the proposed subunit vaccine sequence construct V3.

**Vaccine construct 4:**


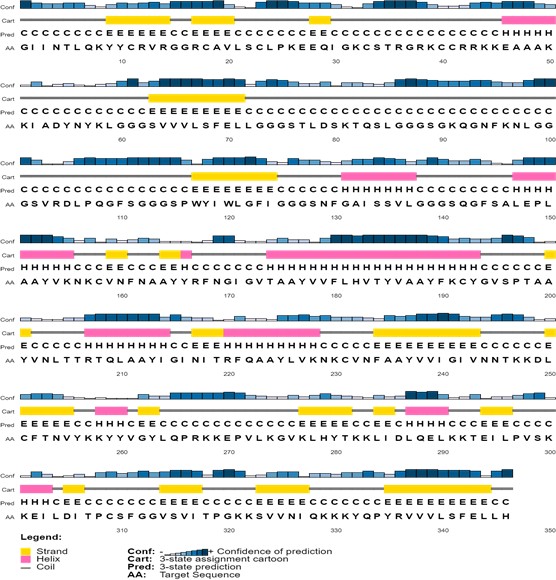


Figure C(iii): Graphical representation of secondary structure features of the proposed subunit vaccine sequence construct V4.

**Vaccine construct V5**


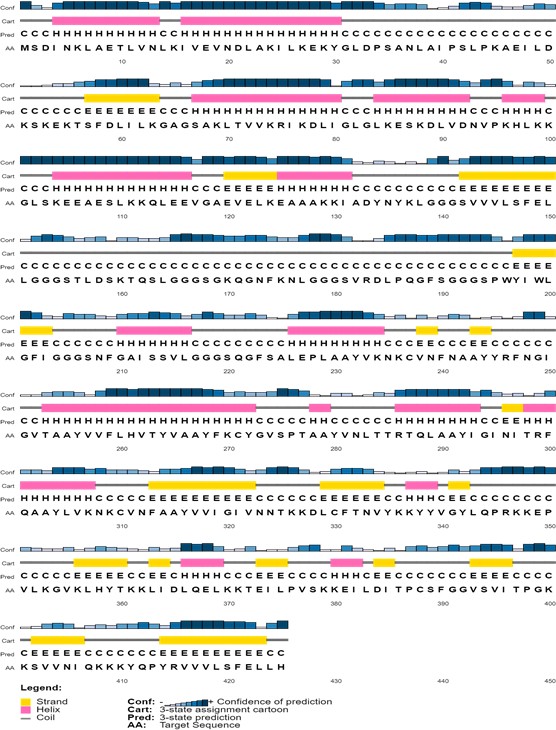


Figure C(iv): Graphical representation of secondary structure features of the proposed subunit vaccine sequence construct V5.

**Vaccine construct V6:**


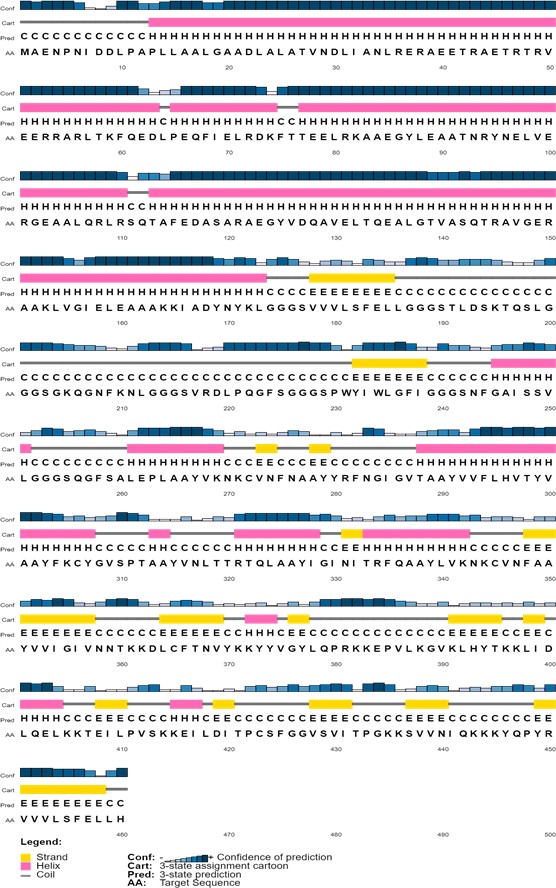


Figure C(v): Graphical representation of secondary structure features of the proposed subunit vaccine sequence construct V6.

Table C: Secondary structure composition of vaccine construct V2-V6 predicted by CFSSP tool

| **Vaccine Construct** | **Percentage composition (%)** | | |
| --- | --- | --- | --- |
|  | **Helix** | **Sheet** | **Turn** |
| **V2** | 73.8 | 68.9 | 11.6 |
| **V3** | 75.0 | 71.6 | 12.3 |
| **V4** | 58.1 | 46.5 | 12.4 |
| **V5** | 69.4 | 64.5 | 11.5 |
| **V6** | 70.9 | 67.4 | 12.2 |

**Supplementary File D**

**Prediction of discontinuous B-cell epitopes**

**Methods:**

Ellipro available at (<http://tools.iedb.org/ellipro/>) (Ponomarenko et al., 2008) was used for identification of conformational epitopes. It assumes that residues protrude from protein surface are more accessible for antibody binding.

**Results:**

Ellipro predicted the four discontinuous B-cell epitopes and confirmed the presence of 210 residues among them with score ranging from 0.60 to 0.98**.**

**Table D**: Discontinuous B-cell epitopes predicted by the ElliPro. Two hundred and ten residues were found to be located in four discontinuous B-cell epitopes of the refined vaccine model.

| **S No.** | **Residues** | **Number of residues** | **Score** |
| --- | --- | --- | --- |
| A | A:G1, A:I2, A:I3, A:N4, A:T5, A:L6, A:Q7, A:K8, A:Y9, A:V13, A:R14 | 11 | 0.98 |
| B | A:Y10, A:C11, A:R12, A:G15, A:G16, A:R17, A:C18, A:A19, A:V20, A:L21, A:S22, A:C23, A:L24, A:P25, A:K26, A:E27, A:E28, A:Q29, A:I30, A:G31, A:K32, A:C33, A:S34, A:T35, A:R36, A:G37, A:R38, A:K39, A:C40, A:C41, A:N69, A:Y70, A:K71, A:L72, A:G73, A:G74, A:G75, A:S76 | 38 | 0.686 |
| C | A:R43, A:E46, A:A47, A:A49, A:K50, A:I52, A:A53, A:D54, A:K58, A:L59 | 10 | 0.668 |
| D | A:Y55, A:N56, A:Y57, A:Y83, A:K84, A:L85, A:G86, A:G87, A:G88, A:S89, A:K90, A:I91, A:A92, A:D93, A:Y94, A:N95, A:K97, A:L98, A:G99, A:G100, A:G101, A:S102, A:K103, A:I104, A:G113, A:G114, A:S115, A:K116, A:I117, A:A118, A:D119, A:Y120, A:N121, A:Y122, A:K123, A:N134, A:K136, A:L137, A:G138, A:G139, A:G140, A:S141, A:K142, A:I143, A:N156, A:K157, A:V159, A:F161, A:N162, A:A163, A:Y177, A:K181, A:C182, A:V183, A:V202, A:K203, A:N204, A:V214, A:K215, A:N216, A:K217, A:C218, A:K227, A:N228, A:K229, A:C230, A:V231, A:N232, A:F233, A:N234, A:A235, A:A236, A:K241, A:C242, A:N246, A:K247, A:C251, A:F252, A:T253, A:N254, A:V255, A:Y256, A:K257, A:D259, A:L260, A:C261, A:F262, A:T263, A:N264, A:V265, A:Y266, A:K267, A:K268, A:D269, A:L270, A:C271, A:F272, A:T273, A:N274, A:V275, A:Y276, A:K277, A:K278, A:D279, A:L280, A:C281, A:F282, A:T283, A:N284, A:V285, A:Y286, A:K287, A:K288, A:D289, A:L290, A:C291, A:F292, A:T293, A:N294, A:V295, A:Y296, A:K297, A:K298, A:D299, A:L300, A:C301, A:F302, A:T303, A:N304, A:V305, A:Y306, A:K307, A:K308, A:D309, A:L310, A:C311, A:F312, A:T313, A:N314, A:V315, A:Y316, A:K317, A:K318, A:D319, A:L320, A:C321, A:F322, A:T323, A:N324, A:V325, A:Y326 | 151 | 0.602 |


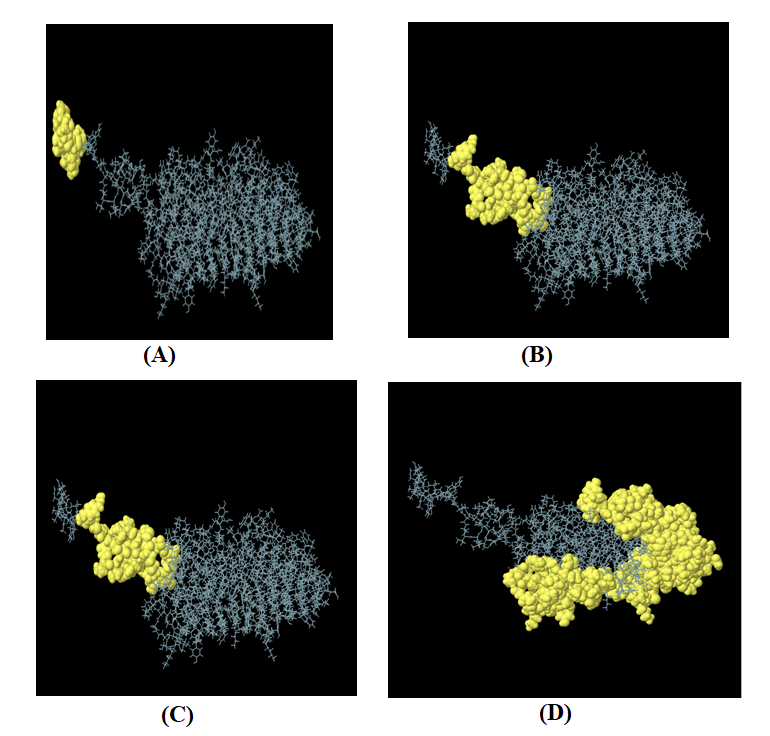


**Figure D**: Discontinuous B-cell epitopes predicted by ElliPro. (a–d): 3D representation of conformational or discontinuous epitopes of the most antigenic chimeric protein. Epitopes are shown as yellow surfaces, and the bulk of the protein is represented in grey sticks

**Reference:**

Ponomarenko, J. *et al.* ElliPro: A new structure-based tool for the prediction of antibody epitopes. *BMC Bioinform.*

**Supplementary File E**

**Viral-Host Protein Interactions:**

The interactions of spike glycoprotein with other host proteins were also investigated using String (v11.0 protein-protein interaction database). Since, at the time of experiment, SARS-CoV-2 interaction data was not available, hence, the data derived from Coronavirus 229E (NCBI taxonomy Id: 11137), Human SARS coronavirus (NCBI taxonomy Id: 694009) and Homo sapiens (host) (NCBI taxonomy Id: 9606) was used. The visual network of viral protein and host protein interaction are shown in Figure D(i) & D(ii). A tabular detail of all the found Human interacting proteins is presented in Table D.

1. A visual network of protein-protein interaction between Human coronavirus 229E proteins (denoted in red) and host Human proteins (denoted in blue).

# Human coronavirus 229E NCBI taxonomy Id: 11137

- - **Homo sapiens NCBI taxonomy Id: 9606**


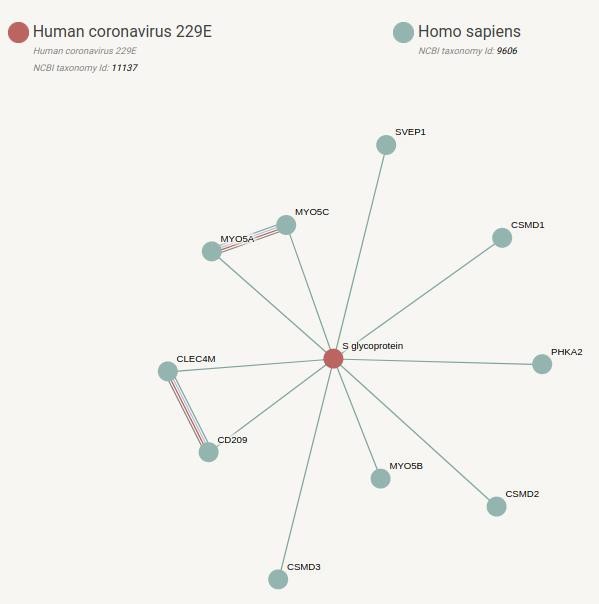


1. A visual network of protein-protein interaction between Human SARS coronavirus (denoted in red) and host Human proteins (denoted in blue).

# Human SARS coronavirus NCBI taxonomy Id: 694009

- - **Homo sapiens (NCBI taxonomy Id: 9606)**


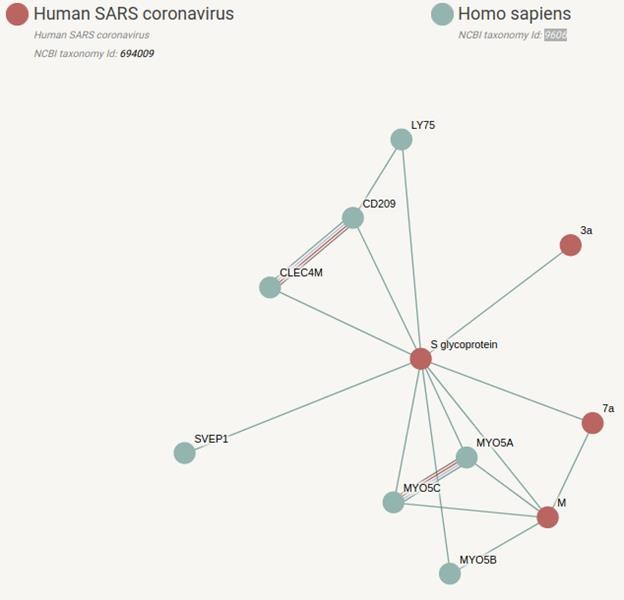


**Table E: Study of protein-protein Interaction of the human host via string v.11 database**

| S.No | Human Interacting Proteins | Scientific Name | Functions | Pathways(Kegg/Gene Card) | Evidence(link) | Summary(Gene card/UniProt) |
| --- | --- | --- | --- | --- | --- | --- |
| 1 | CD209 | Dendritic cell-specific ICAM-3-grabbing non-integrin 1 | Attachment receptor like HIV-1, HIV-2, Ebolavirus, Cytomegalovirus, HCV,Dengue virus, Measles virus, Herpes simplex virus 1,Influenzavirus A, SARS coronavirus, Japanese encephalitis virus, Lassa virus, Respiratory syncytial virus, Rift valley fever virus and uukuniemi virus &West-Nile virus. | - Phagosome (hsa04145) - C-type lectin receptor signalling pathway (hsa04625) - Tuberculosis hsa05152) - Measles (hsa05162) | https://www.nextprot.org/entry/NX_Q9NNX6/ | CD209 (CD209 Molecule) is a Protein Coding gene. Diseases associated with CD209 include Dengue Virus and Human Immunodeficiency Virus Type 1. Among its related pathways are Class I MHC mediated antigen processing and presentation and CLEC7A (Dectin-1) signaling. Gene Ontology (GO) annotations related to this gene include carbohydrate binding and mannose binding. An important paralog of this gene is CLEC4M. |
| 2 | CLEC4M | Dendritic cell-specific ICAM-3-grabbing non-integrin 2 (DC-SIGN2) | Acts as an attachment: receptor:   - Ebolavirus. - Hepatitis C virus. - HIV-1. - Human coronavirus 229E. - Human cytomegalovirus/HHV-5 - Influenza virus. - SARS coronavirus - West-nile virus. Japanese encephalitis virus. - Marburg virus glycoprotein. | - Phagosome (hsa04145) - C-type lectin receptor signalling pathway (hsa04625) - Tuberculosis (hsa05152) - Measles (hsa05162) | https://www.nextprot.org/entry/NX_Q9H2X3/ | CLEC4M (C-Type Lectin Domain Family 4 Member M) is a Protein Coding gene. Diseases associated with CLEC4M include Encephalitis and Japanese Encephalitis. Among its related pathways are Phagosome and C-type lectin receptor signaling pathway. Gene Ontology (GO) annotations related to this gene include calcium-dependent protein binding. An important paralog of this gene is CD209. |
| 3 | MYO5C | Unconventional myosin-Vc | Molecular Function:   - Actin filament binding - Calcium ion binding - Calmodulin binding - Disordered domain specific binding - Identical protein binding - Microfilament motor activity - Protein binding - Rab GTPase binding | Pathogenic Escherichia coli infection (hsa05130) | https://www.nextprot.org/entry/NX_Q9NQX4/ | MYO5C (Myosin VC) is a Protein Coding gene. Diseases associated with MYO5C include Diarrhea 2, With Microvillus Atrophy and Congenital Diarrhea. Among its related pathways are Sertoli-Sertoli Cell Junction Dynamics and Sweet Taste Signaling. Gene Ontology (GO) annotations related to this gene include actin binding and actin filament binding. An important paralog of this gene is MYO5A. |
| 4 | CSMD3 | CUB and sushi multiple domains protein 3 | It is involved in regulation dendrite development | N/A | <https://www.nextprot.org/entry/NX_Q7Z407/> | CSMD3 (CUB And Sushi Multiple Domains 3) is a Protein Coding gene. Diseases associated with CSMD3 include [Benign Adult Familial Myoclonic Epilepsy](http://www.malacards.org/card/benign_adult_familial_myoclonic_epilepsy) and Trichorhinophalangeal Syndrome, Type Ii. An important paralog of this gene is [CSMD1](https://www.genecards.org/cgi-bin/carddisp.pl?gene=CSMD1). |
| 5 | LY75 | Lymphocyte antigen 75 | Acts as an endocytic receptor to direct captured antigens from the extracellular space to a specialized antigen-processing compartment  Molecular function:   - Carbohydrate binding - Signalling receptor activity | Dendritic Cells Development Lineage Pathway | https://www.nextprot.org/entry/NX_O60449/ | LY75 (Lymphocyte Antigen 75) is a Protein Coding gene. Diseases associated with LY75 include [Pneumonic Plague](http://www.malacards.org/card/pneumonic_plague) and [Adenoiditis](http://www.malacards.org/card/adenoiditis). Among its related pathways are [Dendritic Cells Developmental Lineage Pathway](http://pathcards.genecards.org/card/dendritic_cells_developmental_lineage_pathway). Gene Ontology (GO) annotations related to this gene include *carbohydrate binding*. An important paralog of this gene is [LY75-CD302](https://www.genecards.org/cgi-bin/carddisp.pl?gene=LY75-CD302). |
| 6 | MYO5A | Dilute myosin heavy chain, non-muscle | Actin filament binding  ATP binding  Calcium ion binding  Calmodulin binding  Disordered domain specific binding  Identical protein binding  Microfilament motor activity  Rab GTPase binding  RNA binding | Pathogenic Escherichia coli infection | https://www.nextprot.org/entry/NX_Q9Y4I1/ | MYO5A (Myosin VA) is a Protein Coding gene. Diseases associated with MYO5A include [Griscelli Syndrome, Type 1](http://www.malacards.org/card/griscelli_syndrome_type_1_2) and [Griscelli Syndrome, Type 3](http://www.malacards.org/card/griscelli_syndrome_type_3_2). Among its related pathways are [Sertoli-Sertoli Cell Junction Dynamics](http://pathcards.genecards.org/card/sertoli-sertoli_cell_junction_dynamics) and [Translocation of GLUT4 to the plasma membrane](http://pathcards.genecards.org/card/translocation_of_glut4_to_the_plasma_membrane). Gene Ontology (GO) annotations related to this gene include *actin binding*. An important paralog of this gene is [MYO5B](https://www.genecards.org/cgi-bin/carddisp.pl?gene=MYO5B). |
| 7 | PHKA2 | Phosphorylase b kinase regulatory subunit alpha, liver isoform | Phosphorylase b kinase catalyzes the phosphorylation of serine in certain substrates, including troponin I.  The alpha chain may bind calmodulin. | - Calcium signalling pathway (hsa04020) - Insulin signalling pathway (hsa0491) - Glucagon signalling pathway (hsa04922) | <https://www.nextprot.org/entry/NX_P46019/> | PHKA2 (Phosphorylase Kinase Regulatory Subunit Alpha 2) is a Protein Coding gene. Diseases associated with PHKA2 include [Glycogen Storage Disease Ixa1](http://www.malacards.org/card/glycogen_storage_disease_ixa1) and [Glycogen Storage Disease Due To Liver Phosphorylase Kinase Deficiency](http://www.malacards.org/card/glycogen_storage_disease_due_to_liver_phosphorylase_kinase_deficiency). Among its related pathways are [Glycogen Metabolism](http://pathcards.genecards.org/card/glycogen_metabolism) and [Glucose metabolism](http://pathcards.genecards.org/card/glucose_metabolism). Gene Ontology (GO) annotations related to this gene include calmodulin binding and phosphorylase kinase activity. An important paralog of this gene is [PHKA1](https://www.genecards.org/cgi-bin/carddisp.pl?gene=PHKA1). |
| 8 | SVEP1 | Sushi, von Willebrand factor type A, EGF and pentraxin  **or**  Serologically defined breast cancer antigen NY-BR-38 | Play a role in cell attachment process, calcium binding and chromatin binding | N/A | https://www.nextprot.org/entry/NX_Q4LDE5/ | SVEP1 (Sushi, Von Willebrand Factor Type A, EGF And Pentraxin Domain Containing 1) is a Protein Coding gene. Diseases associated with SVEP1 include [Breast Cancer](http://www.malacards.org/card/breast_cancer). Gene Ontology (GO) annotations related to this gene include *calcium ion binding* and *chromatin binding*. An important paralog of this gene is CSMD1. |
