# Supplementary material for: Identification of vaccine targets & design of vaccine against SARS-CoV-2 coronavirus using computational and deep learning-based approaches
Source: PeerJ. 2022 May 19;10:e13380. doi: 10.7717/peerj.13380 (PMC9124463; doi:10.7717/peerj.13380)
Supplement: Supplemental Information 7 [file peerj-10-13380-s007.docx]

**Supplementary File SF**

**Comparison of shortlisted epitopes with epitopes predicted by other tools.**

1. **B-cell epitopes:**

Through our search we identified 3 tools namely, BepiPred 2.0 (Jespersen et al., 2017), ABCpred (Sudipto Saha & Raghava, 2006) and DLBEpitope server (Liu et al., 2020) for prediction of B-cell epitopes. We implemented these tools on the S- Surface glycoprotein [accession no.: QHD43416.1] and obtained epitopes predicted by them. (See **Supplementary SF1-Supplementary_sheet_BCL_epitopes_data**). Further, the predicted epitopes from these tools were subjected to antigenicity prediction using VaxiJen v2.0 server (Doytchinova & Flower, 2007). Their antigenic propensities along with their antigenicity status (Antigen or Non-Antigen) were obtained. Moreover, we have also computed the conservancy of top-most epitope of each tool against 19 different SARS-CoV-2 variants (as mentioned in **section** **2.3** of manuscript) using IEDB Conservancy analysis tool. The topmost epitope of each tool are compared and displayed in Table X1.

**Table X1:** Topmost BCL epitopes of S- Surface glycoprotein [accession no.: QHD43416.1] as predicted by BCEPRED, BepiPred 2.0, ABCpred and DLBEpitope. We observed that the original epitope is overlapping in the topmost epitope predicted by ABCpred and DLBEpitope. The overlapped portion of BCL epitope is highlighted in red.

|  | **BCEPRED (original tool)** | **BepiPred 2.0** | **ABCpred** | **DLBEpitop** |
| --- | --- | --- | --- | --- |
| **Top-most epitope** | DLCFTNVY | GQSKRVDFC | KLNDLCFTNV | KLNDLCFTNV  YADSFVIRGD  EVRQIAPGQT  GKIADYNY |
| **Antigenicity (VaxiJen) score** | 1.8569 | 1.779 | 2.6927 | 1.0205 |
| **Conservancy against 19 SARS-CoV-2 variants** | 100% | 100% | 100% | 97.37-100% |

We observed that Only ABCpred predicted a BCL epitope (KLNDLCFTNV) that has higher antigenicity than the BCL epitope used in vaccine construct (given by BCEPRED). However, on careful observation we found that the amino acid sequence of both the epitopes are aligning/ overlapping. Therefore, we have decided to move forward with the original BCL epitope.

1. **MHC-I or CTL epitopes:**

Similarly, we have identified 5 tools; CTLPred (Bhasin & Raghava, 2004), EpiJen (Doytchinova et al., 2006), NetCTL 1.2 server (Larsen et al., 2007), NetMHCpan-4.1 (Reynisson, Alvarez, et al., 2020) and Tepitool (Paul et al., 2016) for MHC-I (or CTL) epitopes. We have implemented this tool and obtained the predicted epitopes. (See **SF2-Supplementary_sheet_CTL_epitopes_data**, respectively). Their antigenicity and conservancy were evaluated. The topmost epitope of each tools are compared and displayed in Table X2.

**Table X2:** Topmost CTL epitopes of S- Surface glycoprotein [accession no.: QHD43416.1] as predicted by PropredI, CTLpred, EpiJen, NetCTL, NetMHCpan 4.1 and Tepitool.

|  | **PropredI**  **(original tool)** | **CTLpred** | **EpiJen** | **NetCTL 1.2** | **NetMHCpan 4.1** | **Tepitool** |
| --- | --- | --- | --- | --- | --- | --- |
| **Top-most epitope** | KIADYNYKL | TKLNDLCFT | KIADYNYKL | YQDVNCTEV | TKLNDLCFT | TKLNDLCFT |
| **Antigenicity (VaxiJen) score** | 1.6639 | 2.9364 | 1.6639 | 1.6172 | 2.9364 | 2.9364 |
| **Conservancy against 19 SARS-CoV-2 variants** | 88.89-100% | 100% | 88.89-100% | 88.89-100% | 100% | 100% |

Here we observed that top ranking CTL epitope (KIADYNYKL) predicted by EpiJen was same as that of original tool (PropredI). The top ranker epitope predicted by NetCTL 1.2 (YQDVNCTEV) has lower antigenicity than original KIADYNYKL. However, the topmost epitope from all three CTLpred, NetMHCpan and Tepitool tools (TKLNDLCFT) has higher antigenicity and conservancy across 19 variants than the original epitope used in vaccine. However, we have decided to move forward with the original epitope as the epitope TKLNDLCFT belongs to a short peptide segment at location 384-396 on the Spike protein. Coincidentally, our BCL epitope also lies in the same range.

1. **MHC-II or HTL epitopes:**

Three tools were identified for MHC-II epitopes namely, IEDB MHC-II server (Wang et al., 2010), NetMHCIIpan 4.0 server (Reynisson, Barra, et al., 2020) and EpiTOP (Dimitrov et al., 2010). The tools were used to predict HTL epitopes of same protein. Similarly, The antigenicity and conservancy of epitopes were evaluated. (See, **SF3-Supplementary_sheet_HTL_epitopes_data**) The topmost epitope of each tool are compared and displayed Table X3.

Table X3: Topmost HTL epitopes of S- Surface glycoprotein [accession no.: QHD43416.1] as predicted by Propred, IEDB MHC II, NetMHCIIpan 4.0 and EpiTOP.

|  | **Propred (original tool)** | **IEDB MHCII** | **NetMHCIIpan 4.0** | **EpiTOP** |
| --- | --- | --- | --- | --- |
| **Top-most epitope** | VKNKCVNFN | QPYRVVVLSF  ELLHA | PTKLNDLCF | TKLNDLCFT |
| **Antigenicity (VaxiJen) score** | 2.053 | 0.9109 | 2.5304 | 2.9364 |
| **Conservancy against 19 SARS-CoV-2 variants** | 100% | 100% | 100% | 100% |

Next, we are continuing our analysis using original HTL epitope (VKNKCVNFN ) as predicted by Propred since the top ranking epitope predicted by IEDB MHCII server (QPYRVVVLSFELLHA) has lower antigenicity. Although, NetMHCIIpan 4.1 (PTKLNDLCF) and EpiTOP (TKLNDLCFT) predicted epitopes with higher antigenicity, but they lie in the same region as that of BCL epitope i.e., 384-396.

**References:**

1. Bhasin M, Raghava GPS. 2004. Prediction of CTL epitopes using QM, SVM and ANN techniques. Vaccine 22:3195–3204. DOI: 10.1016/j.vaccine.2004.02.005.
2. Dimitrov I, Garnev P, Flower DR, Doytchinova I. 2010. EpiTOP—a proteochemometric tool for MHC class II binding prediction. Bioinformatics 26:2066–2068. DOI: 10.1093/bioinformatics/btq324.
3. Doytchinova IA, Flower DR. 2007. VaxiJen: a server for prediction of protective antigens, tumour antigens and subunit vaccines. BMC Bioinformatics 8:4. DOI: 10.1186/1471-2105-8-4.
4. Doytchinova IA, Guan P, Flower DR. 2006. EpiJen: a server for multistep T cell epitope prediction. BMC Bioinformatics 7:131. DOI: 10.1186/1471-2105-7-131.
5. Jespersen MC, Peters B, Nielsen M, Marcatili P. 2017. BepiPred-2.0: improving sequence-based B-cell epitope prediction using conformational epitopes. Nucleic Acids Research 45:W24–W29. DOI: 10.1093/nar/gkx346.
6. Larsen M V, Lundegaard C, Lamberth K, Buus S, Lund O, Nielsen M. 2007. Large-scale validation of methods for cytotoxic T-lymphocyte epitope prediction. BMC Bioinformatics 8:424. DOI: 10.1186/1471-2105-8-424.
7. Liu T, Shi K, Li W. 2020. Deep learning methods improve linear B-cell epitope prediction. BioData Mining 13:1. DOI: 10.1186/s13040-020-00211-0.
8. Paul S, Sidney J, Sette A, Peters B. 2016. TepiTool: A Pipeline for Computational Prediction of T Cell Epitope Candidates. Current Protocols in Immunology 114. DOI: 10.1002/cpim.12.
9. Reynisson B, Alvarez B, Paul S, Peters B, Nielsen M. 2020a. NetMHCpan-4.1 and NetMHCIIpan-4.0: improved predictions of MHC antigen presentation by concurrent motif deconvolution and integration of MS MHC eluted ligand data. Nucleic Acids Research 48:W449–W454. DOI: 10.1093/nar/gkaa379.
10. Reynisson B, Barra C, Kaabinejadian S, Hildebrand WH, Peters B, Nielsen M. 2020b. Improved Prediction of MHC II Antigen Presentation through Integration and Motif Deconvolution of Mass Spectrometry MHC Eluted Ligand Data. Journal of Proteome Research 19:2304–2315. DOI: 10.1021/acs.jproteome.9b00874.
11. Saha S, Raghava GPS. 2006. Prediction of continuous B-cell epitopes in an antigen using recurrent neural network. Proteins: Structure, Function, and Bioinformatics 65:40–48. DOI: 10.1002/prot.21078.
12. Wang P, Sidney J, Kim Y, Sette A, Lund O, Nielsen M, Peters B. 2010. Peptide binding predictions for HLA DR, DP and DQ molecules. BMC Bioinformatics 11:568. DOI: 10.1186/1471-2105-11-568.

**Comment 4:**

I do not understand the purpose of “3.5 Randomisation experiments”. The authors first need to explain the need for such an analysis in their work. The authors only mentioned during this analysis that the score of epitopes from the shuffled protein sequence was significantly different from the score of epitopes predicted from the original protein sequence. This is so much crude. What was like the numerical values of such scoring? How far it was statistically significant in terms of any P-values OR confidence interval etc.

**Response:**

Thank you. As per your suggestion, we have removed this section.

**Minor Comments**

**Comment 1:**

Figure 1 is quite basic and need to be discarded.

**Response:**

Thank you. As per the recommendation, we have modified the figure 1 in the revised version. The new figure denotes the components of SARS-CoV-2 along with Spike protein receptor binding site, Angiotensin-converting enzyme 2 (ACE2) and Transmembrane serine Protease TMPRSS2.

**Comment 2:**

Figure 3; the A, B, C and D should be explained separately in the figure legend.

**Response:**

In the revised manuscript the figure 3 is now figure 4.

**Earlier caption of figure 3:**

Figure 3: Protein-peptide docking using web server HPEPDOCK showing peptide (I-1V from MHC-I glycoprotein) in golden yellow colour with HLA-A*02 protein. The above diagram represents the protein-peptide docking with the top-4 selected MHC-I epitope sequences with Human Leukocyte Antigen protein.

A: KIADYNYKL B:VVVLSFELL C:TLDSKTQSL D:GKQGNFKNL

**Modified caption in new manuscript of figure 4:**
